# Supplementary material for: Emergence of two distinct regimes in phonon-induced non-equilibrium magnetization dynamics
Source: Newton. 2026 Jun 1;2(6):None. doi: 10.1016/j.newton.2026.100509 (PMC13226452; doi:10.1016/j.newton.2026.100509)
Supplement: Document S1. Figures S1–S11, Table S1, and Notes S1–S8 [file mmc1.pdf]

NEWTON, Volume *2*

Supplemental information

Emergence of two distinct regimes in phonon-induced non-equilibrium magnetization dynamics

Jim Groefsema, Viktoriia Radovskaia, Thom Janssen, Nils Dessmann, Vladislav Bilyk, Peter K. Kim, Timur T. Gareev, Meng Xing Na, Jorrit R. Hortensius, Andrea D. Caviglia, Theo H.M. Rasing, Andrei I. Kirilyuk, Carl S. Davies, Alexey V. Kimel, and Dmytro Afanasiev

Note S1. The crystal structure of $DyFeO_3$

In Fig. S1 we present the crystal structure of $DyFeO_3$ using the space group *Pnma*. The structure is of an orthorhombically distorted perovskite. The unit cell consists out of 4 formula units, where octahedrons of oxygen atoms encase the iron atoms [1,2].

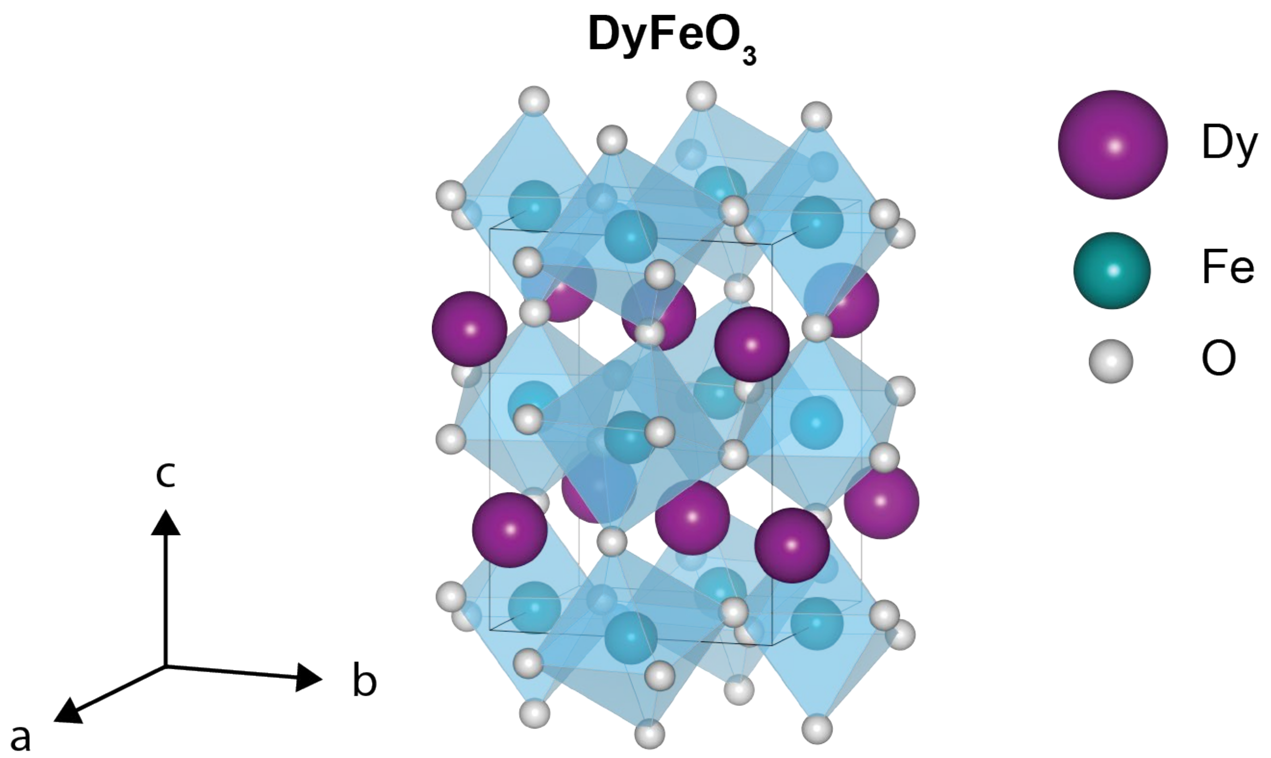

Figure S1 | Crystal structure of $DyFeO_3$. The crystal structure of $DyFeO_3$. The purple, green and white sphere represent dysprosium, iron and oxygen atoms respectively. The oxygen octahedra are shaded in blue.

Note S2. Fitting the FTIR spectrum

The full reflectivity spectrum of $DyFeO_3$ obtained through Fourier-transform infrared spectroscopy (FTIR) can be found in Fig. S2.

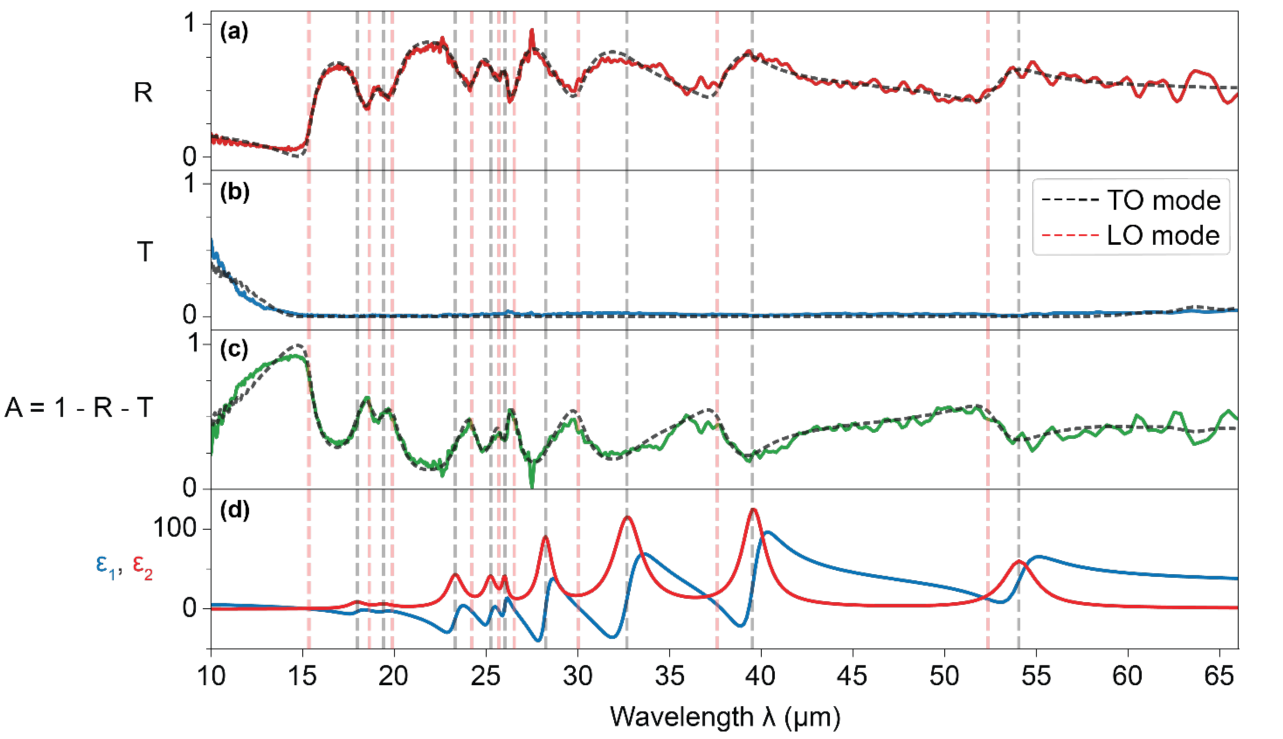

Figure S2 | The reflectivity spectrum of $DyFeO_3$. The infrared reflectivity **(a)** and transmission **(b)** spectrum of $DyFeO_3$ through Fourier-transform infrared spectroscopy (FTIR) obtained at room temperature. The absorption **(c)** is obtained through the relation $A = 1 - R - T$. The black dashed lines are the resulting spectrum obtained after fitting the data using the Drude-Lorentz model. The FTIR measurement was taken using unpolarized light. The angle of incidence used is 30°. **(d)** The calculated elements of the complex dielectric permittivity $\tilde{\epsilon}(\omega) = \epsilon_1(\omega) + i\epsilon_2(\omega)$ where $\epsilon_1(\omega)$ and $\epsilon_2(\omega)$ are the real and imaginary parts of $\tilde{\epsilon}(\omega)$ respectively.

We obtained the complex dielectric permittivity, $\tilde{\epsilon}(\omega)$ by simultaneously fitting the reflectivity and transmission spectrum to Drude-Lorentz oscillators (Eq. S2.1) and the Fresnel equations. The light source used was unpolarized and at an angle of incidence of 30°, so to describe the reflectivity and transmission data, we can take the average between *p*- and *s*-polarized light, detailed in equations S2.2 and S2.3:

$$\tilde{\epsilon}(\omega) = \epsilon_1(\omega) + i\epsilon_2(\omega) = \epsilon_\infty + \sum \frac{S_i \omega_{Tj}^2}{\omega_{Tj}^2 - \omega^2 - i\omega\gamma_j}, \qquad (S2.1)$$

$$\langle R \rangle = |\langle r \rangle|^2 = \left|\frac{r_p + r_s}{2}\right|^2 = \left|\frac{\sin^2\alpha\,(\tilde{\epsilon}(\omega) - 1)}{\left(\cos\alpha + \sqrt{\tilde{\epsilon}(\omega) - \sin^2\alpha}\right)\left(\tilde{\epsilon}(\omega)\cos\alpha + \sqrt{\tilde{\epsilon}(\omega) - \sin^2\alpha}\right)}\right|^2 \qquad (S2.2)$$

$$\langle T \rangle = \left|(1 - \langle r \rangle^2)\, e^{i\frac{\omega}{c}\sqrt{\tilde{\epsilon}(\omega)}d}\right|^2 \qquad (S2.3)$$

where ϵ_∞ is the high-frequency value of the dielectric permittivity, $\epsilon_1(\omega)$ and $\epsilon_2(\omega)$ are the real and imaginary parts of the dielectric permittivity respectively, ω_{Tj}, γ_j, and S_j are the frequency, damping, and oscillator strength of a given transverse optical (TO) phonon mode, c

is the speed of light, d the sample thickness, and r_p and r_s are the reflectance coefficients for p- and s-polarized light at an angle of incidence α. ϵ_∞ is obtained by fitting the model up to 7000 cm^{-1}. To see how well this fitting procedure follows the reflectivity spectrum, Fig. S2A and B shows both the experimentally found data points and the resulting fit using Eq. S2.1. Fig. S2C shows the absorption spectrum for both the data points and the resulting fit obtained through the relation $A = 1 - R - T$, where A is the absorption, R the reflectivity and T the transmission. Fig. S2D shows the resulting dielectric permittivity, decomposed into the real part ϵ_1 and the imaginary part ϵ_2 as obtained through the Drude-Lorentz fitting procedure.

Nine Drude-Lorentz oscillators were identified and fitted. The parameters used for the fitting procedure can be found in table S1. Using the connection $\omega = \frac{2\pi c}{\lambda}$, where c is the speed of light and λ the wavelength, we can write ω_T and ω as λ_T and λ respectively. $\epsilon_\infty$was found to be 7.8 by fitting the high frequency part of the FTIR data. We compared the results to the phonon mode frequencies reported in Ref [3], but since we used unpolarized light to capture the reflectivity spectrum, the phonon symmetry mode cannot be accurately determined. As a result, the phonon modes in table S1 are only a suggestion for the potential mode that is resonant at this wavelength.

Phonon mode $\lambda_\#$	λ_T (μm)	S	Y (μm)	Suggested phonon mode	DFT λ_T (μm)
9	54.06	2.32	1371.55	$B_{3u}(2)$	53.49
8	39.58	4.67	1036.01	$B_{1u}(3)$	39.99
7	32.70	6.34	582.36	$B_{3u}(5)$	32.36
6	28.23	2.67	902.54	$B_{2u}(5)$	28.49
5	26.01	0.36	2100.79	-	-
4	25.23	1.00	861.75	$B_{1u}(7)$	25.45
3	23.31	1.81	522.69	$B_{3u}(7)$	23.58
2	19.44	0.28	321.13	$B_{3u}(8)$	19.45
1	17.96	0.47	300.45	$B_{1u}(9)$	18.42

Table S1 | $DyFeO_3$ reflectivity fitting parameters. The calculated parameters of the transverse optical (TO) phonon modes. The parameters are λ_T, S and γ, which represent the wavelength, oscillator strength and damping of a given TO phonon mode. Nine modes have been found and described. A suggestion of the potential phonon mode along with its calculated wavelength is given using the DFT calculations as reported in Ref. [3].

Figure S3 shows the schematic of every modelled phonon eigenmode as found in Table S1. The lattice distortions correspond to a stretching of the oxygen bonds, displayed as yellow arrows.

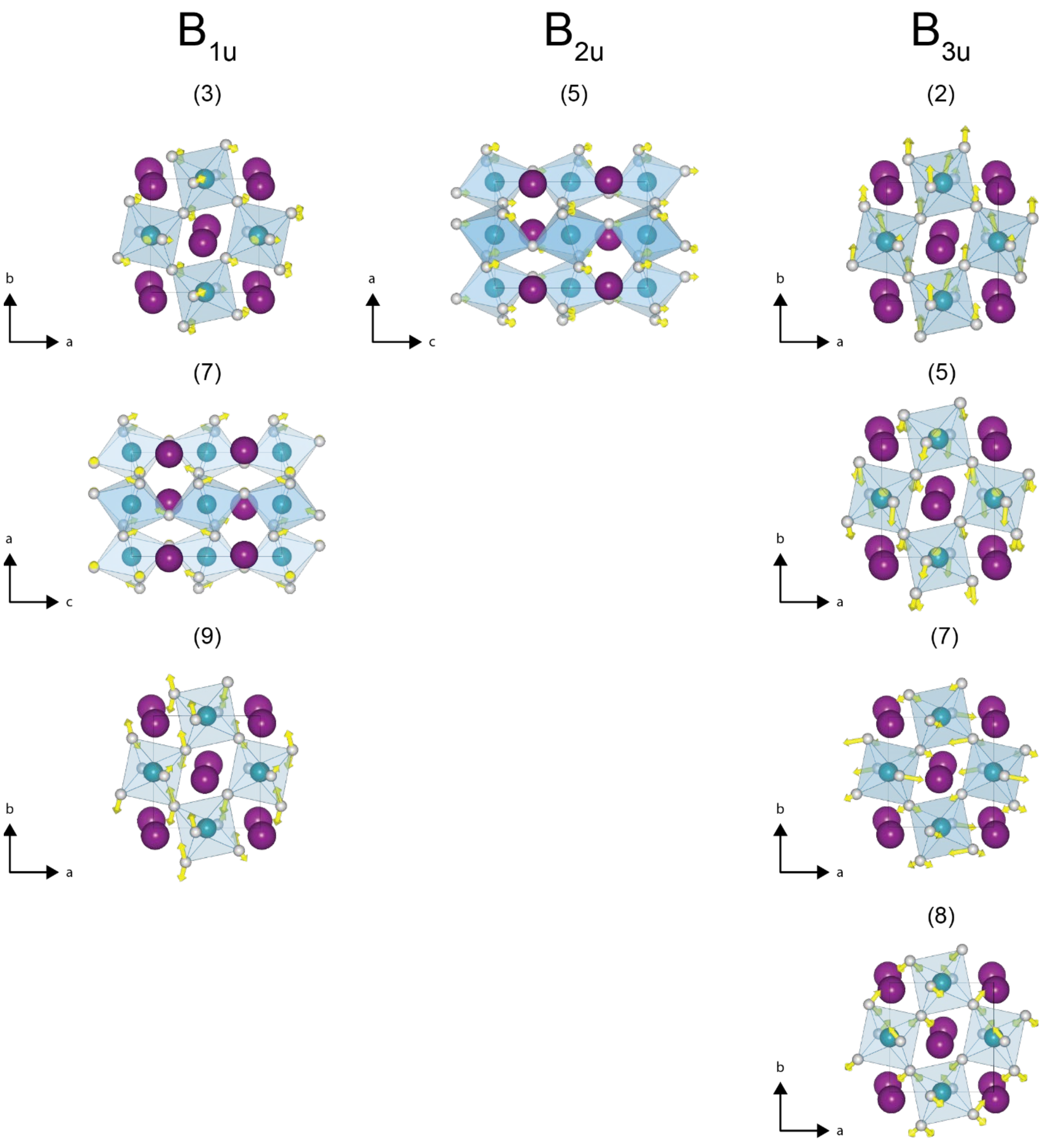

Figure S3 | Calculated phonon eigenmodes. Eight phonon eigenmodes are displayed here. Three corresponding to the B_{1u} mode, one to the B_{2u} mode and four to the B_{3u} mode. The purple/blue/gray spheres correspond to dysprosium, iron and oxygen atoms. The oxygen octahedra are shaded in light blue. Yellow arrows show the movement caused by the phonon eigenmode. The bracketed numbers correspond to the degeneracy as found in Ref [3].

Note S3. Fluence dependence of the macropulse measurement at different wavelengths

We measured the fluence dependence to determine the critical fluence above which the PT is triggered. To obtain a threshold fluence F_c at which a phonon-induced transition occurs, we first assume a Gaussian fluence distribution for our laser pulse,

$$F = F_0\, e^{\frac{-x^2}{2\sigma^2}}, \qquad (S3.1)$$

where F_0 is the peak fluence, x is a spatial coordinate in the plane of the sample, and σ the beam diameter. The Gaussian profile has been illustrated in Fig. S3.1a. We can define a critical threshold fluence, F_c as the fluence at the boundary of the phonon-induced WFM state observed through MO imaging at $x_c \equiv d/2$, where d is the diameter. Thus we can write,

$$d = 2\sigma\sqrt{2\ln\left(\frac{F_0}{F_c}\right)}. \qquad (S3.2)$$

We experimentally measure the fluence dependence of the diameter of phonon-induced state at various wavelengths as shown in Fig. S3.1b. We can fit the data to Using Eq. S3.2, to extract a critical threshold fluence for each wavelength used, F_c. These fit values for $1/F_c$ are shown in Fig. 5a as stars.

Next, to fit the measured diameter data d at constant fluence F_0 and varying wavelength, as seen in Fig. 5a, Eq. S3.2 was used where $1/F_c$ is modeled as a sum of two Gaussian functions with two distinct maxima at the wavelengths λ_{max}=14 μm and λ_{max}=18.5 μm in accordance with our findings from the fluence dependence measurement. In this fitting procedure we have taken σ to be 354 μm as obtained through the fitting procedure of the fluence dependence and F_0 has been kept constant at 91 mJ/cm^2. The two summed Gaussian functions can be written as the following,

$$\mathrm{F_c}(\lambda) = \alpha_1 e^{-\frac{(\lambda-\mu_1)^2}{2\rho_1{}^2}} + \alpha_2 e^{-\frac{(\lambda-\mu_2)^2}{2\rho_2{}^2}}, \qquad (S3.3)$$

where $\alpha_{1,2}$ is the inverse of the threshold fluence F_c at resonance, λ is the pump wavelength, $\mu_{1,2}$ resonance wavelength and $\rho_{1,2}$ the spectral width of the F_c peaks. Considering the agreement of the fitting results using Eq. S3.2 and Eq. S3.3 with the diameter data, we can conclude that the nature of the phonon-induced PT necessitates a critical fluence to occur that is strongly wavelength dependent with two clear resonances at λ_{max}=14 μm and λ_{max}=18.5 μm.

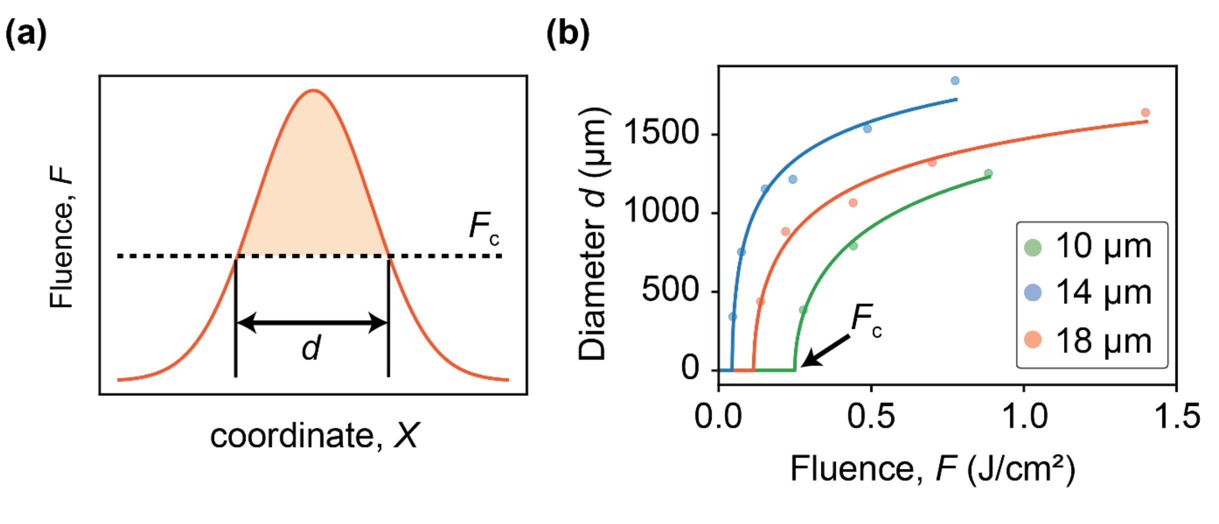

Figure S4 | Fitting the fluence dependence. (a) The spatial Gaussian distribution of the fluence of the pump pulse. The dashed line represents the critical fluence F_c above which the photo-induced phase transition occurs. The diameter d is defined here as the edge of the coordinate X where the phase transition is visible. **(b)** The diameter d of the photo-induced WFM region as a function of the mid-infrared (MIR) pump fluence in J/cm^2. The solid line is a logarithmic fit for the data points as described in Eq. (S3.2). The value at which d reaches zero is marked by an arrow and referred to as the critical fluence F_c. Three pump wavelengths are shown, marked by their own separate color.

Note S4. Phononic contributions to the absorption spectrum

To further understand the contribution of each phonon mode to the absorption spectrum, Fig. S5 presents each phonon mode in terms of their absorption using the Drude-Lorentz model and Eqs. S2.2 and S2.3. We see that depending on the strength of the oscillator, we can obtain significant absorption "wings" stretching out several micrometers beyond its resonance wavelength. A splitting occurs in the absorption due to the LO-TO splitting, creating a Reststrahlen band in between. The indices of every mode seen in Fig. S5 corresponds to the phonons with the same labels in Fig. 5 and table S1.

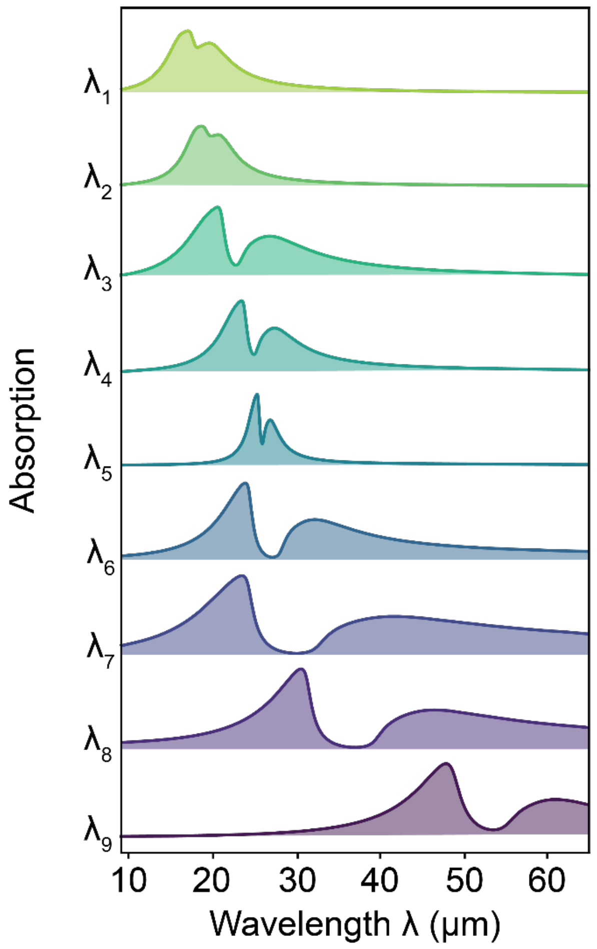

Figure S5 | Phonon contributions to the absorption model. (a) The absorption spectrum for every calculated phonon mode is shown separately with a differently colored shaded curve.

Note S5. Fluence dependence of the micropulse

Our imaging experiments reveal that the micropulse fluence dependence closely follows that observed in the macropulse regime, exhibiting a clear threshold behaviour (see Fig. S6). When the fluence exceeds this threshold, the area undergoing the phase transition increases, resulting in a larger overall magnetization change.

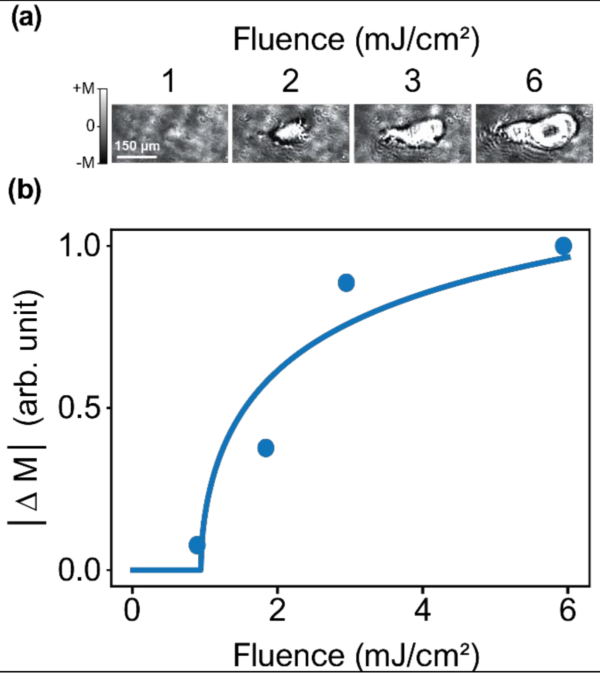

Figure S6 | Micropulse fluence dependence (a) Magneto-optical images of the phonon-induced spot using micropulses in a single-shot regime at various given fluences. **(b)** The change in magnetization as a function of the micropulse fluence. A blue curve is given as a guide to the eye.

To estimate how the lifetime of the nonthermal state evolves as a function of fluence, we present in Fig. S7 the magnetization dynamics extracted from vertical slices of the MO images at different distances from the beam center. Assuming a Gaussian excitation profile, the position of each slice provides an estimate of the local pump fluence. Using this approach, we determine the lifetime of the non-thermal process, $\tau_{non\text{-}th}$, as a function of the pump fluence, showing that the lifetime of the nonthermal, phonon-driven process is only weakly dependent on the pump fluence, see Fig. S7C. At higher pump fluences, $\tau_{non\text{-}th}$ falls slightly compared to the lower fluences, following the lifetimes of the WFM precession modes [4], known to be governed by the spin-lattice interaction.

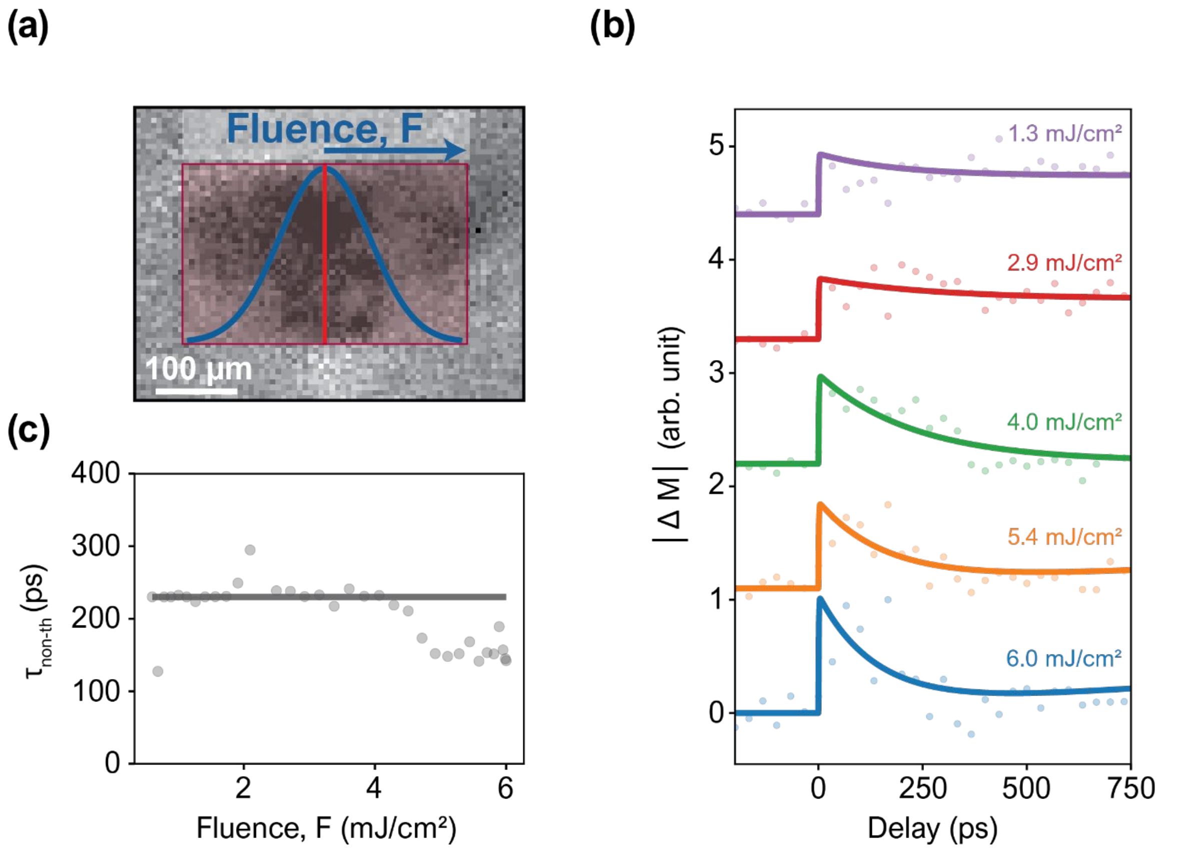

Figure S7| Micropulse fluence dependence through image slices. (a) An example of the method used to extract the dynamics at different fluences. A pixel wide slice (red vertical line) of a magneto-optical image is taken and compared to differing time delays. The horizontal position of the slice dictates the local fluence that was experienced on the sample through a Gaussian distribution (with the blue curve provided as a guide to the eye). **(b)** The magnetization dynamics given through the vertical slices in the magneto-optical images at different time delays. A three exponential fit is provided as a solid curve for each fluence. **(c)** The lifetime of the non-thermal process as a function of the determined fluence. A grey horizontal line is given as a guide to the eye.

Note S6. Extended Polarization Control

Regarding the polarization angles where ΔM is close to zero (e.g., around 165°), a coexistence of two largely homogeneous domains with opposite magnetization orientations is observed at early delays, consistent with the absence of an energetic preference for either state. This behaviour is consistent with our nonthermal model: at these polarizations, the coupling to the two energetically equivalent magnetization states is similar, leading to their equal population and thus a near-zero net magnetization. As shown in Fig. S8, over time the initially large nonthermal domains begin to fragment, giving rise to smaller 'black' and 'white' domains, which signals a gradual crossover toward a thermal, multidomain state.

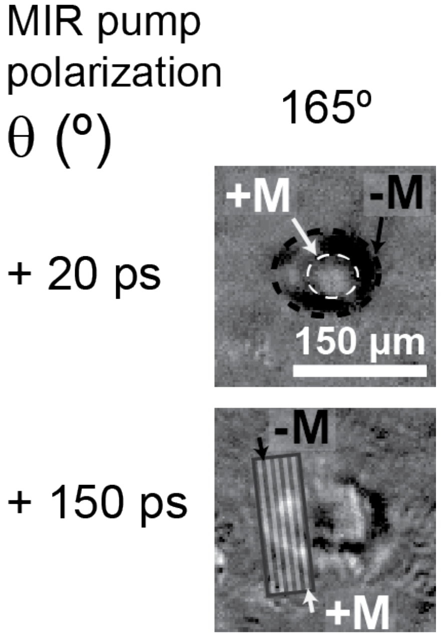

Figure S8 | Pump polarization dependence The pump polarization is here described as the angle θ between the crystallographic a-axis and the impulse light's polarization. Two sets of magneto-optical images were taken, one at a delay of 20 ps after the arrival of the pump beam and another at 150 ps. At 20 ps two homogeneous domain are highlighted. A stripe pattern that emerges at 150 ps is displayed.

When phonon pumping in $DyFeO_3$, coherent spin precession are additionally excited [3]. To understand how the pump polarization can affect these spin precessions, we performed additional tabletop stroboscopic pump–probe measurements to clarify which pathway governs the phonon-driven response. As shown in Fig. S9, the pump polarization modifies the amplitude of the spin precession, which shows some correlation with the polarization dependence of the phonon-induced magnetization. However, it does not reverse the precession phase. Therefore, while an anisotropic contribution from the precession may be present, it does not appear to set the state selection.

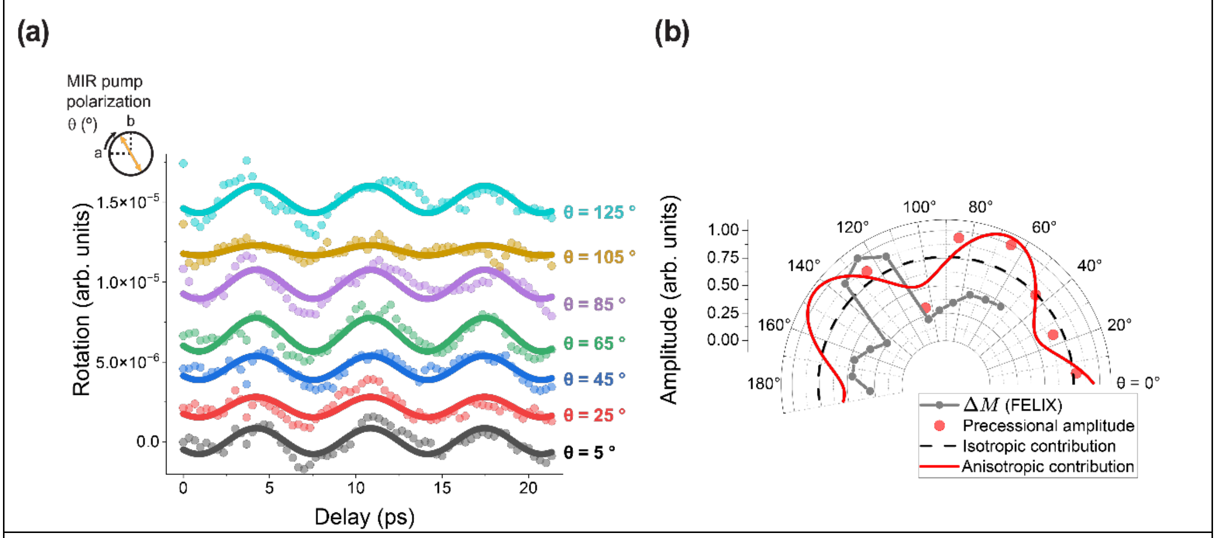

Figure S9 | Polarization dependent spin precession (a) The resulting pump induced spin precession observed through the magneto-optical time-resolved Faraday effect extracted after an exponential fit. The solid curves are sine wave fits performed along the relevant data points for a given pump polarization θ. **(b)** The amplitude of the sine fits performed in **(a)** are presented along with the given pump polarization θ. The isotropic contribution is displayed as the black curve, while an additional sine fit is given to highlight its anisotropic behavior. The sample was held at the AFM phase with a temperature of 34 K while an external magnetic field of 20 mT was applied. The pump wavelength used was 14 µm. Additionally, grey data points show the change in magnetization through the phonon-induced phase transition, where now an amplitude of 0 represents the black magnetization state and an amplitude of 1 represents the white magnetization state seen previously through magneto-optical imaging.

Note S7. Wavelength dependence between the macro- and micropulse

In Fig. S10B the wavelength dependence of the phonon-induced phase transition is presented for both the macropulse regime (Fig. S10A) as well as the micropulse regime (Fig. S10C). Both wavelength dependencies agree on the position of λ^{max}, where the resonance peaks at λ^{max} = 14 µm and λ^{max} = 18.5 µm for both regimes.

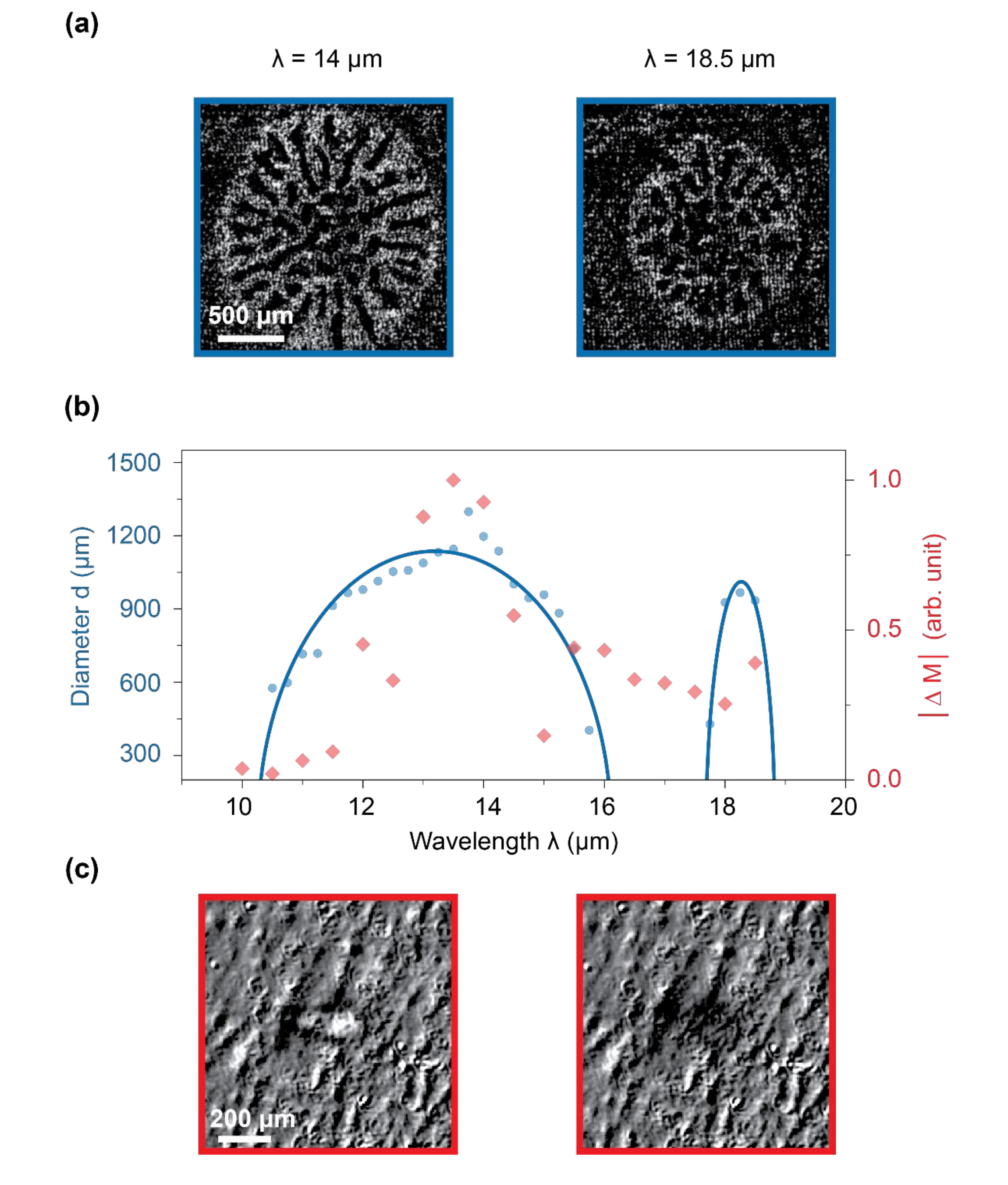

Figure S10 | Ultrafast dynamics induced by a single MIR micro-pulse at 18.5 µm. A comparison between the wavelength dependence using the macropulse (**(a)** and **(b)** left, blue dots), measured using the diameter of the switched spot, and the micropulse (**(c)** and **(b)** right, red diamonds), measured using the integrated intensity. The blue curve is the fitting procedure performed on the macropulse results. Two sets of magneto-optical (MO) images highlight the peak amplitude from the graphs at $\lambda = 14$ µm and $\lambda = 18.5$ µm. **(a)** The blue border signifies the macropulse regime while **(c)** the red border represents the micropulse regime. The micropulse MO images are taken 90 ps after the pump pulse.

Note S8. Ultrafast dynamics induced by a single MIR micro-pulse at 18.5 µm

While our results in the main text focused on the dynamics at 14 µm due to the stronger response, Fig. S11 shows that the ultrafast dynamics are similar to Fig. 3 for the phonon resonance found at 18.5 µm. The shape of the photo-excited spot appears to be highly elliptical as it forms, shown in Fig. S11a. This can be due to the shape of the elliptical

micropulse itself at this wavelength. Despite the shape of the spot, the dynamics remain largely unchanged, as Fig. S11B and Fig. S11C shows. The initial rise of the magnetization is on a similar timescale as seen in Fig. 4b at $\tau_{rise} = 7$ ps.

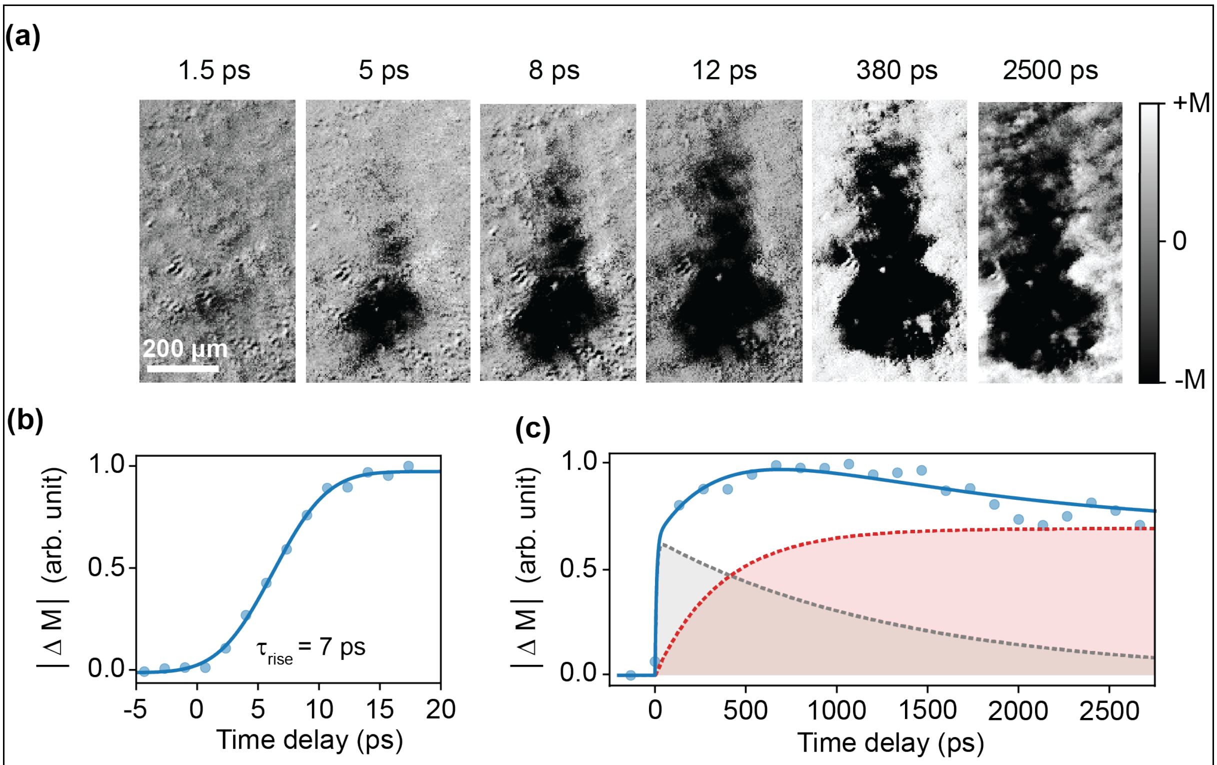

Figure S11 | Ultrafast dynamics induced by a single MIR micro-pulse at 18.5 µm. (a) Magneto-optical images are shown at different time delays to highlight the development of the Morin phase transition (PT) when pumping at 18.5 µm from an initial temperature of 46 K. **(b)** The magnetization dynamics are shown, where the normalized magnetization is plotted against the time delay in ps after the pump beam up to 20 ps. The solid line represents a fit using an error function. **(c)** The magnetization dynamics of the Morin PT is shown by displaying the normalized magnetization as a function of the time delay up to 2700 ps. The solid blue line in the graph is a fit with three exponentials representing the initial rise, exponential decay, and a slower exponential rise. The dotted red line is the part of the fit that highlights the thermal contribution, and the dotted gray line is the non-thermal contribution to the fit.

We performed the same fitting procedure for the dynamics up to 2.7 ns and found that we can identify the three processes seen in Fig. 3c: the initial fast rise, the exponential decay, and the slower exponential rise. The timescale for the exponential decay, however, is not on the same order as the slower exponential rise, suggesting that the nonthermal component remains present for the duration of the time interval measured. One reason for this discrepancy can be due to a lack of data points supporting this process in comparison to the results seen in Fig. 3.

References

1. Cui, R., Jiang, H., Du, Y., Xu, Y., Jia, Y., Sun, K., and Hao, X. (2024). Role of Dy *4f* electrons on magnetic coupling and reorientation in DyFeO3. J. Phys.: Condens. Matter *36*, 335501. https://doi.org/10.1088/1361-648X/ad49f9.

2. Hoogeboom, G.R., Kuschel, T., Bauer, G.E.W., Mostovoy, M.V., Kimel, A.V., and van Wees, B.J. (2021). Magnetic order of Dy^{3+} and Fe^{3+} moments in antiferromagnetic $DyFeO_3$ probed by spin Hall magnetoresistance and spin Seebeck effect. Phys. Rev. B *103*, 134406. https://doi.org/10.1103/PhysRevB.103.134406.

3. Afanasiev, D., Hortensius, J.R., Ivanov, B.A., Sasani, A., Bousquet, E., Blanter, Y.M., Mikhaylovskiy, R.V., Kimel, A.V., and Caviglia, A.D. (2021). Ultrafast control of magnetic interactions via light-driven phonons. Nat. Mater. *20*, 607–611. https://doi.org/10.1038/s41563-021-00922-7.

4. Balbashov, A.M., Volkov, A.A., Lebedev, S.P., Mukhin, A.A., and Prokhorov, A.S. (1985). High-frequency magnetic properties of dysprosium orthoferrite. Journal of Experimental and Theoretical Physics *88*, 974–987.
