# Supplementary material for: Emergence of two distinct regimes in phonon-induced non-equilibrium magnetization dynamics
Source: Newton. 2026 Jun 1;2(6):None. doi: 10.1016/j.newton.2026.100509 (PMC13226452; doi:10.1016/j.newton.2026.100509)
Supplement: Document S2. Article plus supplemental information [file mmc2.pdf]

Newton

Article

Emergence of two distinct regimes in phonon-induced non-equilibrium magnetization dynamics

Graphical abstract

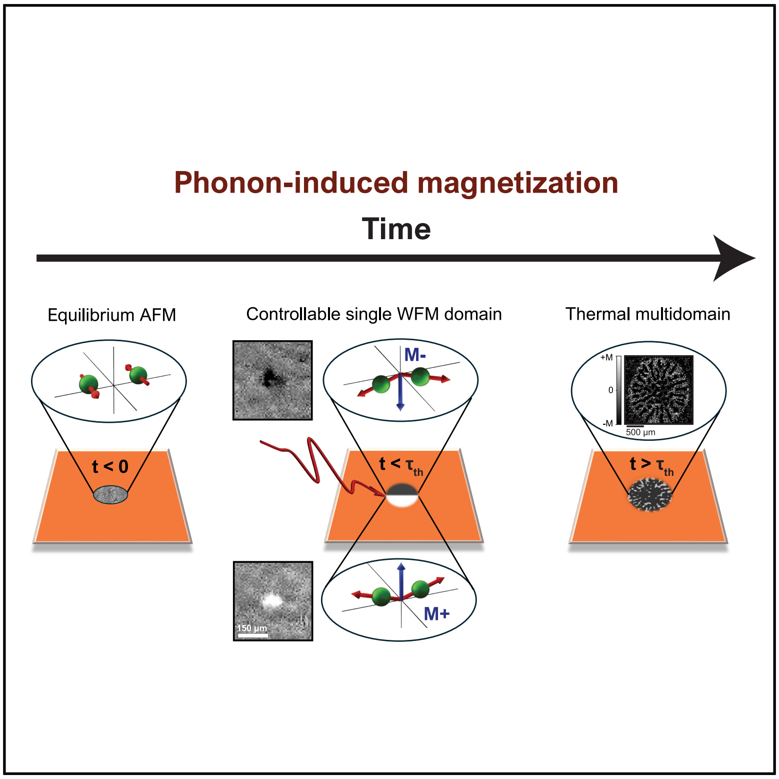

Highlights

- Resonant MIR phonon excitation induces non-thermal magnetic phase transition in 10 ps
- Single-shot magneto-optical imaging reveals non-thermal-to-thermal crossover in 230 ps
- Polarization of MIR light pulse controls direction of non-thermal-induced magnetization
- Drude-Lorentz model demonstrates excitation through multiple IR-active phonons

Authors

Jim Groefsema, Viktoriia Radovskaia, Thom Janssen, ..., Carl S. Davies, Alexey V. Kimel, Dmytro Afanasiev

Correspondence

jim.groefsema@ru.nl (J.G.),
d.afanasiev@science.ru.nl (D.A.)

In brief

Non-linear phononics can be used to manipulate magnetic materials on short timescales. Groefsema et al. use ultrafast mid-infrared laser pulses in $DyFeO_3$ to drive a transition from an antiferromagnetic to a weakly ferromagnetic state. They visualize the crossover from a non-thermal, phonon-driven magnetization to a thermal multidomain state occurring after 200 ps. This exceeds the phonon lifetime and matches the spin–lattice relaxation time, revealing how lattice vibrations can be harnessed to manipulate magnetic order and define fundamental limits for ultrafast control.

Groefsema et al., 2026, Newton 2, 100509
June 1, 2026 © 2026 The Author(s). Published by Elsevier Inc.
https://doi.org/10.1016/j.newton.2026.100509

CellPress

Newton

CellPress
OPEN ACCESS

Article

Emergence of two distinct regimes in phonon-induced non-equilibrium magnetization dynamics

Jim Groefsema,[1,6,*] Viktoriia Radovskaia,[1] Thom Janssen,[5] Nils Dessmann,[5] Vladislav Bilyk,[1] Peter K. Kim,[1] Timur T. Gareev,[1] Meng Xing Na,[1] Jorrit R. Hortensius,[2,3] Andrea D. Caviglia,[4] Theo H.M. Rasing,[1] Andrei I. Kirilyuk,[5] Carl S. Davies,[5] Alexey V. Kimel,[1] and Dmytro Afanasiev[1,*]

[1]Institute for Molecules and Materials, Radboud University, 6525 AJ Nijmegen, the Netherlands

[2]Kavli Institute of Nanoscience, Delft University of Technology, P.O. Box 5046, 2600 GA Delft, the Netherlands

[3]Electromagnetic Signatures and Propagation, TNO, 2597 AK The Hague, the Netherlands

[4]Department of Quantum Matter Physics, Université de Genève, 24 Quai Ernest-Ansermet, 1211 Geneva, Switzerland

[5]HFML-FELIX Laboratory, Radboud University, 6525 ED Nijmegen, the Netherlands

[6]Lead contact

*Correspondence: jim.groefsema@ru.nl (J.G.), d.afanasiev@science.ru.nl (D.A.)

https://doi.org/10.1016/j.newton.2026.100509

ACCESSIBLE OVERVIEW Lattice excitations are typically viewed as incoherent and disruptive, akin to heating that destroys magnetic order. However, recent progress has shown that when specific lattice vibrations, or phonons, are resonantly excited with ultrafast mid-infrared laser pulses, they can coherently distort the crystal structure and induce non-equilibrium states that are inaccessible by conventional heating. This approach, called non-linear phononics, has recently emerged as a powerful tool for controlling electronic and magnetic properties on extremely short timescales. While such lattice-driven control has already enabled remarkable phenomena, it has remained unclear how long these non-equilibrium states persist before relaxing back to thermal equilibrium. In this work, we investigate this question in the magnetic material dysprosium orthoferrite ($DyFeO_3$). By selectively exciting phonon vibrations with intense mid-infrared laser pulses, we trigger a transition from an antiferromagnetic state to a weakly ferromagnetic state. Using a specialized imaging technique that captures snapshots of the magnetic state after each laser pulse, we track how this transition unfolds in both time and space. Immediately after excitation, the material enters a non-thermal regime characterized by a uniform magnetization whose direction is set by the polarization of the infrared light, even in the absence of an applied magnetic field. At later times, the magnetization reorganizes into a multidomain pattern that no longer depends on the laser polarization, signaling that the system has relaxed toward thermal equilibrium. We quantify the crossover between these regimes, which occurs after 200 ps and is consistent with the characteristic spin-lattice relaxation time of the weakly ferromagnetic phase. By directly visualizing this transition, our results provide new insight into how lattice vibrations can be used to manipulate magnetic order and reveal fundamental limits for ultrafast control of materials.

SUMMARY

Driving infrared (IR)-active phonons to large amplitudes to enable non-equilibrium crystal lattice distortions, known as non-linear phononics, can initiate phase transitions along non-thermal pathways, providing transient control of various material properties beyond the equilibrium limits. Yet, how these non-thermal lattice-driven states evolve and thermalize remains unresolved. Here, we explore the crossover from non-thermal to thermal magnetization dynamics in dysprosium orthoferrite ($DyFeO_3$), driven by the resonant excitation of IR-active phonons. Using mid-infrared light pulses, we induce a transition from the collinear antiferromagnetic to the weakly ferromagnetic (WFM) phase, resulting in the emergence of net magnetization. Time-resolved single-shot magneto-optical imaging across multiple timescales reveals two distinct regimes. First, magnetization emerges as a spatially uniform state whose direction is controlled by the pump polarization, indicative of a non-thermal mechanism driven by non-linear phononics. On longer timescales, this state relaxes into a multidomain pattern that is insensitive to the pump polarization, consistent with thermal

© 2026 The Author(s). Published by Elsevier Inc.
This is an open access article under the CC BY license (http://creativecommons.org/licenses/by/4.0/).

equilibration. The crossover occurs on a timescale of about 200 ps, far exceeding the IR phonon coherence time and consistent with the spin-lattice relaxation time in the WFM phase. These findings provide direct temporal and spatial fingerprints of non-linear-phononics-driven magnetic phase control, defining intrinsic limits for reversible ultrafast manipulation of magnetic order.

INTRODUCTION

The development of table-top and free-electron laser (FEL) sources capable of delivering ultrashort and intense optical pulses in the mid-to far-infrared spectral range has opened new pathways for controlling the quantum properties of materials. These pulses can be tuned to resonantly excite infrared (IR)-active lattice vibrations, also known as phonons. If the vibrational amplitudes become sufficiently large, non-linear interactions can couple the driven phonon modes to other, previously inaccessible phonon modes—a process known as non-linear phononics. This provides coherent lattice distortions that are unattainable in equilibrium by tuning parameters such as pressure or strain and can induce states not represented in the thermodynamic phase diagrams. The distortions can break particular crystal symmetries and thereby have dramatic effects on electronic and magnetic properties of media, offering a means for ultrafast control of functional phases and material properties. Phenomena such as lattice-driven spin switching,[1,2] induction of ferroelectric polarization,[3–7] metal-insulator phase transitions,[8,9] and the emergence of room-temperature superconductivity[10] were reported and have become subjects of intense debates. Furthermore, recent works indicate that optically driven phonons can lead to the emergence of magnetization even in materials with no pre-existing spin structure, an effect called dynamic multiferroicity.[11–14]

Phase transitions (PTs) driven by non-linear phononics often proceed through non-thermal pathways, such as transient reshaping of the free-energy landscape or renormalization of interactions.[4,15] As a result, conventional quasi-thermal models based on effective temperatures break down.[15] Although these transitions eventually thermalize and relax into phases consistent with the equilibrium thermodynamic phase diagram, many studies have shown that states created through non-linear phononics can persist for surprisingly long times, exceeding 1 ns,[16–19] despite the rapid decay of the resonantly driven IR-active phonon coherence within just a few picoseconds. This anomalously long-lived behavior raises fundamental questions about the origin of such metastability alongside the mechanisms that govern the stability and eventual thermalization of these transient states.

The rare-earth antiferromagnetic perovskite dysprosium orthoferrite ($DyFeO_3$) provides an ideal platform to experimentally study the crossover from non-thermal, non-linear-phononics-driven magnetization dynamics to the thermally driven regime, enabling direct comparison of these two distinct pathways within a single material. Recent work has demonstrated that resonant phonon pumping in $DyFeO_3$ can induce a transition from the low-temperature collinear antiferromagnetic (AFM) phase to the high-temperature weakly ferromagnetic (WFM) phase,[20] resulting in the emergence of net magnetization. Notably, although the WFM phase is thermally accessible via population of the rare-earth 4f states following both below- and above-band-gap electronic excitation,[3,20] the non-linear-phononics-driven phase transition proceeds through a fundamentally different, non-thermal pathway. This pathway involves phonon-driven distortions of the crystal lattice that strongly modify the Fe-Dy exchange coupling, directly driving the phase transition and inducing a dramatic, long-lived reconfiguration of the magnetic energy landscape that is not accessible via thermal manipulation and occurring on a timescale significantly faster than that of the thermal process.[20] Although the non-thermal nature of the phonon-induced magnetization dynamics has been well established, the timescales and mechanisms underlying the subsequent thermalization remain unclear.

In this study, we employ single-shot pump-probe magneto-optical (MO) imaging at the FEL facility FELIX in Nijmegen, the Netherlands, to investigate the crossover from non-linear-phononics-driven to heat-driven magnetization dynamics and identify temporal and spatial fingerprints of this transition. Using high-intensity, tunable, and ultrashort mid-infrared (MIR) pulses from the FEL, we selectively drive two distinct IR-active phonon modes in $DyFeO_3$, inducing a transition from the AFM to the WFM phase. We capture MO snapshots of the magnetization dynamics across multiple timescales, from picoseconds to hundreds of microseconds, revealing two distinct regimes of magnetization behavior. At short timescales, the magnetization dynamics are spatially homogeneous, with its orientation controlled by the pump polarization. At longer timescales, the magnetization becomes spatially inhomogeneous, forming a multidomain pattern that is largely insensitive to the pump polarization. We attribute these changes to the thermalization of the system, governed by the characteristic spin-lattice relaxation time in the WFM phase of $DyFeO_3$.

RESULTS

Magnetic and lattice structure of $DyFeO_3$

$DyFeO_3$ has an orthorhombically distorted perovskite structure of the space group D_{2h}^{16}-*Pnma*[21,22] with four formula units per unit cell (Figure S1). The magnetism of $DyFeO_3$ originates from the magnetic iron (Fe^{3+}) ions that are antiferromagnetically aligned below the Néel temperature ($T_N = 645$ K) and undergo a spin reorientation transition (SRT) at $T_M \approx 51$ K.[21] Below T_M, Fe^{3+} spins align antiparallel with respect to the *b* axis, and above T_M, the spins reorient along the *a* axis while developing a small mutual canting toward the *c* axis. This SRT—widely known as the Morin transition by analogy to a similar transition found in antiferromagnetic hematite (α-Fe_2O_3)—leads to a PT from a collinear AFM phase with no net magnetization into a canted WFM phase characterized by a finite net magnetization *M* along the *c* axis.

The Morin phase transition is of first order, leading to a competition of the AFM and WFM phases in the vicinity of T_M where both phases are simultaneously present in the phase diagram.[23] The net magnetization in the WFM phase, combined with a

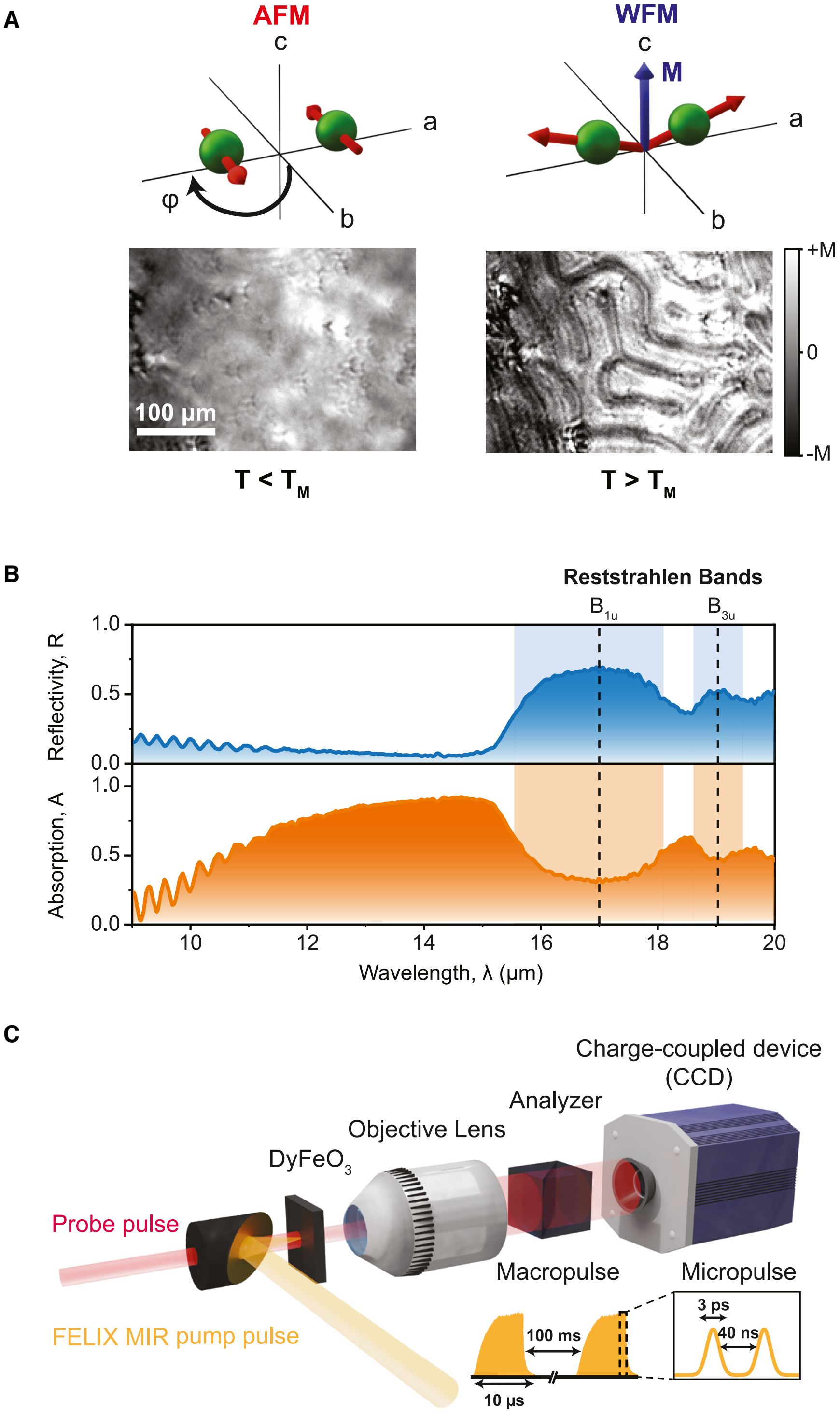

Figure 1. The magnetic phases of $DyFeO_3$ and experimental setup

(A) Magneto-optical images, showing the magnetization change across the Morin transition ($T_M \approx 51$ K) in $DyFeO_3$. Left: the low-temperature antiferromagnetic (AFM) phase. Right: domain pattern formation in the weakly ferromagnetic (WFM) phase. A schematic of the spin orientation in each phase is displayed above. The red arrows are spins, and the blue arrow highlights the net magnetization in the WFM phase.

(B) The infrared reflectivity spectrum of $DyFeO_3$ obtained through Fourier transform infrared (FTIR) spectroscopy (in blue). The absorption of $DyFeO_3$ is obtained through FTIR measurements through the relationship $A = 1 - R - T$ as the orange curve. A shaded region shows the Reststrahlen bands present in the data.

(C) Schematic of the pump-probe imaging setup. The mid-infrared (MIR) pump beam (yellow) induces the AFM-to-WFM phase transition in $DyFeO_3$. An objective lens and a charge-coupled device (CCD) are placed behind the $DyFeO_3$ sample to capture magneto-optical images generated by the probe pulse (red). The MIR pump pulse from the FEL comes as an 8-μs burst ("macropulse") of temporally shorter pulses with a width of 3 ps at a spacing of 40 ns.

strong MO response, allows for the visualization of the competing magnetic AFM and WFM phases using MO microscopy.[24,25] Figure 1A shows MO images of magnetic phases in a c-cut thin slab of $DyFeO_3$ below (left) and above (right) T_M along with the corresponding configurations of the Fe^{3+} spins. While we do not resolve the magnetic domain pattern in the collinear AFM phase due to a lack of net magnetization, a distinct pattern of worm-like domains emerges in the canted WFM phase. The pattern consists of quasiperiodic "dark" and "bright" stripes, corresponding to regions with M aligned along or opposite to the c axis.

The lattice structure of $DyFeO_3$ hosts a series of IR-active phonon modes with B_{1u}, B_{2u}, and B_{3u} symmetry.[20,26] The coupling between the lattice vibrations and incident electromagnetic radiation results in broad Reststrahlen bands, spectral regions of high reflectivity, in the MIR spectral range (Figure S2). This coupling also leads to pronounced absorption features in $DyFeO_3$ spanning a wavelength, λ, from approximately 10 to 100 μm. Figure 1B displays the short-wavelength region of the unpolarized Fourier transform infrared (FTIR) reflectivity spectrum $R(\lambda)$ of a c-cut $DyFeO_3$ sample, revealing distinct and well-defined Reststrahlen bands of B_{1u} and B_{3u} phonons centered at around 17 μm and 19 μm. The corresponding absorption bands, calculated as $A(\lambda) = 1 - R(\lambda) - T(\lambda)$ with $T(\lambda)$ denoting the transmission spectrum, are also shown and appear at slightly shorter wavelengths of 14 μm and 18.5 μm.

MIR pulses from the FELIX facility were employed to resonantly excite IR-active phonon modes and induce a net

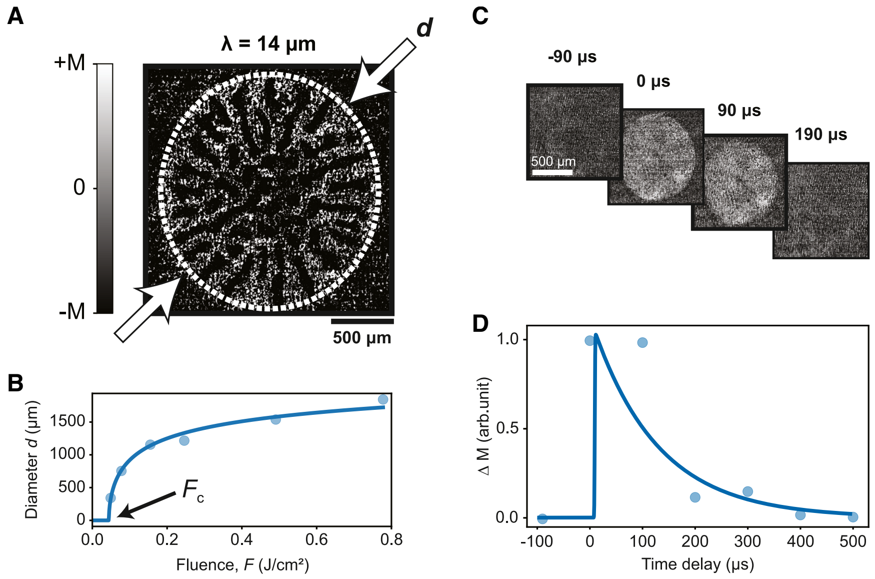

Figure 2. Macropulse-induced magnetization in $DyFeO_3$

(A) A magneto-optical image taken at 44 K, 90 μs after the photo-induced Morin phase transition in $DyFeO_3$ is created by a pump pulse at 14 μm. The diameter *d* of the photo-induced WFM spot in the image is highlighted with a white dashed ellipse.
(B) The diameter *d* of the photo-induced WFM region as a function of the mid-infrared (MIR) pump fluence in J/cm^2. The solid line is a logarithmic fit for the data points as described in Note S3. The value at which *d* reaches zero is marked by an arrow and referred to as the critical fluence F_c.
(C) Magneto-optical images at several time delays. A magnetic field along the *c* axis was applied to force a monodomain state. The indicated region of interest (the images) is used to calculate the magnetization.
(D) Relaxation of the photo-induced magnetization in $DyFeO_3$. The initial temperature of the sample is 40 K. The pump is tuned to be resonant to the phonon mode at 18.5 μm. The blue curve is a fit of an exponential decay. At zero time delay, the rapid development of the photo-induced phase transition was not distinguishable in the macropulse regime, and the rise has been assumed to be instantaneous (<1 μs).

magnetization in $DyFeO_3$ (Figure 1C). These pump pulses are delivered in the form of 8-μs-long bursts ("macropulses") at a repetition rate of 10 Hz, with each macropulse comprising several hundred transform-limited MIR pulses ("micropulses") spaced 40 ns apart. To capture the dynamics of the induced magnetization, two types of time-resolved MO imaging experiments were conducted. In the first macropulse scheme, the sample is pumped by a single macropulse containing approximately 200 micropulses, and magneto-optically imaged by a continuous-wave (CW) laser that is measured by an electronically triggered charge-coupled device (CCD) camera. In the second micropulse scheme, the pump beam consists of a single micropulse sliced from the macropulse[27] and is probed magneto-optically using a synchronized Ti:sapphire laser pulse.

Imaging the thermally driven Morin phase transition on the microsecond timescale

Figure 2A presents an MO snapshot of the sample 90 μs following exposure to a single MIR macropulse, with a central wavelength λ = 14 μm. This wavelength lies near the onset of the high-reflectivity region and matches the peak of the shortest-wavelength absorption band as shown in Figure 1B. In the absence of an applied magnetic field, the linearly polarized MIR excitation induces a spatially inhomogeneous pattern of worm-like magnetic domains with opposite magnetizations, shown in black and white. This pattern resembles the multidomain state observed in the equilibrium WFM phase. However, unlike the equilibrium state, where the domains form stripe-like structures aligned along the crystallographic *c* axis, here the photoexcited region adopts a radial pattern spreading outward from the pump center and remains confined within a well-defined circular region. We note that the domain pattern emerges only above a well-defined macropulse fluence threshold of F_c^M = 44 mJ/cm^2, beyond which its diameter, *d*, gradually increases with higher fluences (Figure 2B and Note S3).

To estimate the lifetime of the lattice-driven magnetic state, we recorded the MO images as a function of time delay from the macropulse. We employed an external magnetic field (100 Oe) applied along the *c* axis to eliminate the multidomain pattern, resulting in a monodomain state where the magnetization aligns with the field's direction (Figure 2C) to reduce the complexity of the pattern. The value of the magnetization is estimated by taking the integrated intensity of the MO image within a region of interest (ROI). The MO images seen in Figure 2C are examples of the size of the ROI used in this case. By tracking the magnetization as a function of the time delay between the macropulse and the CCD's exposure, we observe that the magnetization dynamics decay exponentially on a timescale of 100 μs, after which the system recovers to its initial AFM state (Figure 2D).

This timescale suggests that the recovery is governed by thermal diffusion. Furthermore, we observe that the pump-induced multidomain structure (Figure 2A) shows no sensitivity to the pump polarization angle or its helicity. Together, these observations indicate that the macropulse-induced state likely corresponds to a quasi-steady magnetic state formed via cumulative heat-driven excitation: the approximately 200 micropulses, separated by only 40 ns, do not allow the system to fully relax between pulses, leading to a gradual temperature buildup that drives the sample into a weakly ferromagnetic state without a preferential orientation in the absence of an external magnetic field.

Imaging the non-thermally driven Morin phase transition on the picosecond timescale

While the magnetization induced by the full 8-μs-long macropulse suggests thermalization, we contrast this to the dynamics

CellPress
OPEN ACCESS

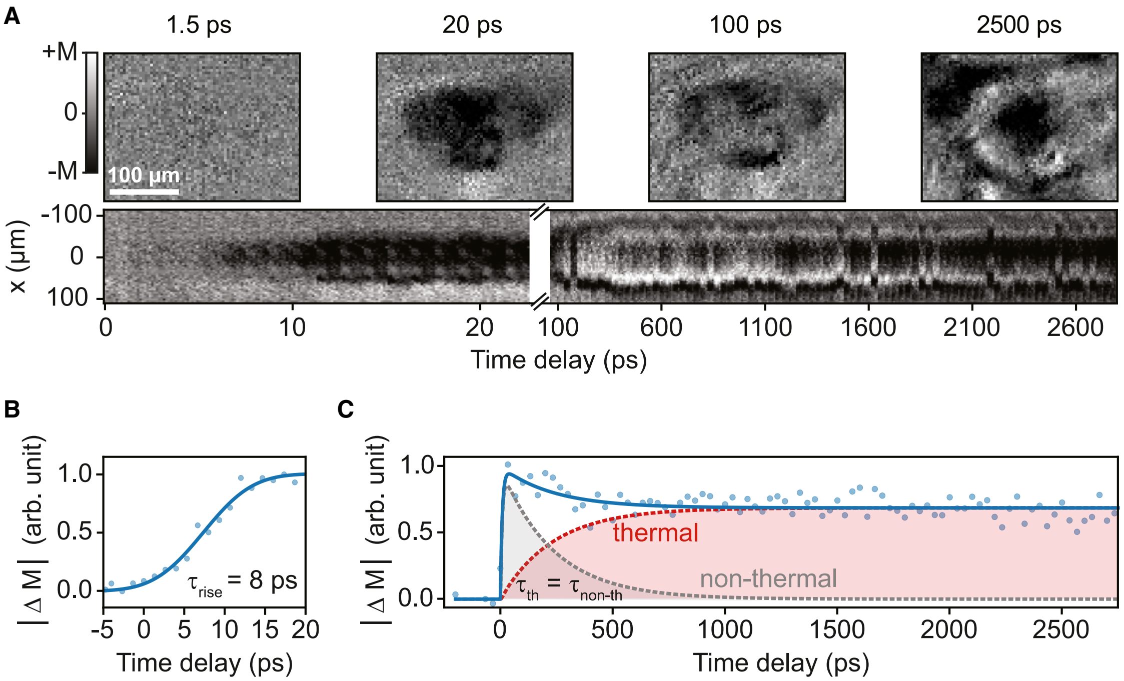

Figure 3. Ultrafast magnetization dynamics driven by a single micropulse

(A) Cross-section of the phonon-induced area stretched over time. The two resulting magnetization states are represented in black and white, with a lack of magnetization shown as gray. On the left, the slices are shown up to 20 ps. On the right, the longer timescales are represented, up to 2.7 ns. Magneto-optical images are shown at different time delays to highlight the development of the PT with a temperature of 46 K. These images also serve as examples of the region of interest used.

(B) Magnetization dynamics of the PT, plotted against the time delay up to 20 ps. The solid line represents a fit using an error function.

(C) Magnetization dynamics of the PT shown by displaying the normalized net magnetization as a function of the time delay up to 2,700 ps. The solid blue line in the graph includes three exponential fits. One fit simulates the initial rise of the magnetization. A dotted red line is the part of the fit that highlights the thermal contribution; the dotted gray line is the non-thermal contribution to the fit.

on the picosecond timescale, which exhibit distinctly non-thermal behavior. Previous stroboscopic pump-probe studies have shown that resonant excitation of the same phonon band with ultrashort pulses can trigger non-thermal magnetization on a picosecond timescale.[20] To observe a crossover between thermal and non-thermal behaviors, we isolate a single micropulse from the macropulse and capture the corresponding single-shot MO response under zero applied magnetic field. Here, the time resolution is limited by the duration of the micropulse ($\sim$3 ps). The micropulses are tuned to the wavelength λ = 14 µm, the same as the macropulse experiment. As in the macropulse case, fluences above threshold lead to expansion of the phase-transitioned region (Note S5). Figure 4A shows temporally and spatially resolved MO snapshots of ultrafast switching up to 2.7 ns under these above-threshold conditions. There are several features in the ultrafast dynamics that strikingly deviate from observations using macropulse excitation. First, the magnetization generated by a single picosecond-long micropulse exhibits a high degree of spatial homogeneity, particularly during the initial phase, lasting few hundreds of picoseconds, of rapid intensity and diameter growth. In contrast to the multidomain pattern seen under macropulse excitation, the magnetization here displays a round-shaped homogeneous domain, even at zero applied magnetic field. This results in a transient net magnetization, not present in the equilibrium phase diagram, indicating that the dynamics are following a non-thermal pathway. At a later time, the spot begins to develop signs of spatial inhomogeneity characterized by the appearance of white domains within the black spot (right panel of Figure 3A).

The time-evolution of the net magnetization, ΔM, is shown in Figures 3B and 3C. By fitting the ΔM dynamics, we identify three distinct processes: an initial rapid rise (Figure 3B), a subsequent decay (Figure 3C, gray curve), and a slower, gradual increase at later times (Figure 3C, red curve). The fast onset of the phonon-driven magnetization within $\sim$8 ps is inconsistent with thermal mechanisms following above- or below-band-gap electronic pumping, which are limited by weak spin-lattice coupling of the 4f rare-earth ions and therefore unfold on much longer timescales of order 100 ps.[3] Instead, the observed sub-10-ps growth suggests that the phase transition proceeds via a non-thermal, non-linear phononics-driven pathway, agreeing with previous stroboscopic studies.[20] In this scenario, a phonon-induced distortion modifies the magnetic energy landscape and launches a coherent spin precession with an amplitude large enough to overcome the energy barrier and switch between the competing AFM and WFM phases. Although the single-shot nature and the length of the micropulse in our experiment prevent direct observation of the spin precession reported by Afanasiev et al.,[20] the

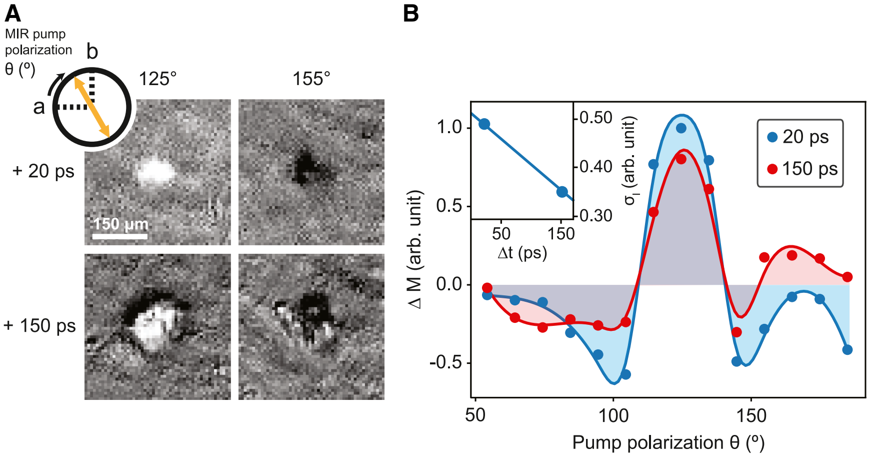

Figure 4. Polarization-defined control of the magnetic state

(A) Pump polarization defined as the angle between the crystallographic *a* axis and the light polarization, highlighted by θ. Two magnetization orientations are presented using two different angles of θ. The magneto-optical (MO) images in which both states can be observed are presented. These images were taken 20 ps after the pump beam hit the $DyFeO_3$ sample. Two other MO images were taken at 150 ps after the arrival of the pump beam to display the transition to the inhomogeneous magnetic state.

(*B*) Normalized magnetization as a function of the pump polarization, θ. A guide to the eye is included for 20 ps (blue) and 150 ps (red). To illustrate the degree of control, two shaded regions from zero to its given value along the guide to the eye is presented for both 20 ps in blue and 150 in red, with the gray region representing an overlap in both time delays. An inset shows the standard deviation σ_l of the normalized magnetization from zero. A linear extrapolation highlights the downward trend of σ_l.

measured rise time closely matches the characteristic period of the precession, lending support to this non-thermal scenario. As Figure 3C shows, the exponential decay and rise dynamics that follow the initial rise are characterized by a strikingly similar characteristic timescale $\tau_{th} \simeq \tau_{non\text{-}th} \simeq 230$ ps. These two concurrent and competing processes are accompanied by the emergence and growth of oppositely oriented magnetic domains from an initially uniform magnetization pattern (Figure 3A), marking the transition from non-thermal, phonon-driven dynamics to thermally driven processes.[20,28] Our experiments indicate that the lifetime of the non-thermal, phonon-driven process is only weakly dependent on the applied pump fluence (Note S5). The crossover timescale falls squarely within the range of spin-lattice relaxation times reported for $DyFeO_3$ in the WFM phase.[20,28] Since the spin-lattice relaxation differs markedly between the AFM and WFM phases and is strongly influenced by the interaction between the rare-earth and iron sublattices, the close correspondence with the WFM relaxation time suggests that the thermalization of the phonon-induced magnetization is governed by spin-lattice coupling in this phase. In this context, the large angular momentum of the Dy^{3+} ions and their strong exchange coupling to the Fe sublattice might imply that the Dy^{3+} moments act as an angular-momentum reservoir, prolonging the relaxation of the transient non-thermal WFM state well beyond the lifetime of the pumped phonon mode. The resulting multidomain WFM phase then gradually relaxes back to the AFM ground state over ~100 µs via thermal diffusion, as shown by our macropulse measurements.

To further explore the non-thermal nature of the phonon-induced states, we investigate whether the orientation of the induced magnetization can be controlled by the polarization of the micropulse. Figure 4A shows that even without an applied magnetic field, the net magnetization ΔM can be controlled by rotating the MIR micropulse polarization relative to the crystallographic *a* and *b* axes. At 20 ps, the spatially homogeneous "black" domain, indicating a net magnetization pointing into the sample plane, changes to a spatially homogeneous "white" domain, indicating a net magnetization pointing out of the sample plane, when the polarization of the micropulse is altered. At later times, the degree of control over the net magnetization state deteriorates and signs of an inhomogeneous multidomain state appear.

The polarization-controlled phase switching observed in our experiments is a clear hallmark of a non-thermal process, as it cannot be attributed solely to polarization-independent absorption of the pump. Together with dynamics that are markedly faster than those driven by ultrafast heating,[3] this provides an additional signature of the non-thermal character of the phonon-driven mechanism. These observations, combined with the previously reported red shift of the phonon-driven magnon frequency below its equilibrium value, indicative of a softening of the magnetic potential,[20] strongly support this conclusion. Moreover, the gradual loss of pump-polarization dependence at longer time delays not only serves as an additional signature of the crossover from a non-thermal to a thermal state but also allows us to quantify the timescale over which this crossover occurs.

We quantify the loss of pump-polarization dependence by integrating the intensity of the MO image at each angle θ, which defines the orientation of the pump-polarization plane with respect to the crystal's *a* axis, as illustrated in Figure 4B. While the polarization dependence is non-trivial, owing to the strong birefringence of $DyFeO_3$ that complicates a direct mapping between the external pump polarization and the crystal axes, it nevertheless demonstrates that a magnetization state ΔM with a well-defined orientation can be selectively prepared during the non-thermal regime. We also note that at angles for which ΔM approaches zero, a coexistence of two largely homogeneous domains with opposite magnetization orientations is observed, consistent with the absence of an energetic preference for either state (Note S6). Polarization control of the coherent phonon-induced precession in $DyFeO_3$ is a prime candidate to explain the sensitivity of the magnetization sign.[3] However, our stroboscopic table-top experiments reveal that the pump polarization modulates the amplitude of the induced spin precession but does not reverse its phase (Figure S9). Since

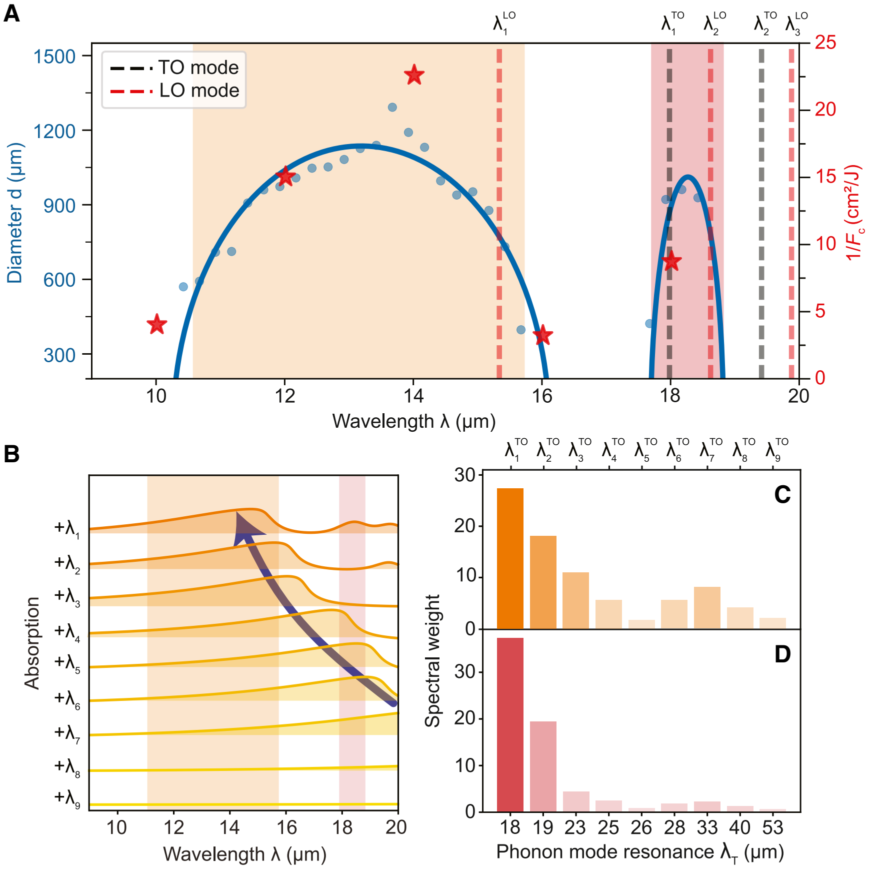

Figure 5. Resonant enhancement of phonon-induced magnetization

(A) Measured diameter d of the phonon-induced spot. The blue curve is included as a fit (see Note S3). The pump fluence for each macropulse is 91 mJ/cm^2. The MO images in which the diameter was measured were taken 90 μs after the pump pulse. Red stars point toward the inverse of the critical fluence F_c. Throughout (A), two types of dashed lines are included to show the behavior of the transverse optical (TO) phonons (black) and longitudinal optical (LO) phonons (red). Relevant regions of high absorption within the material are shown through two shaded regions, in orange and red.

(B–D) (B) A set of absorption spectra where modeled phonon modes are activated in sequence, starting from the highest resonance wavelength mode λ_T. Each absorption spectrum is accompanied by a shaded region to define its spacing from zero absorption. The labels next to the curve specify which mode has been activated. The two shaded regions along all absorption spectra represent the relevant high absorption as seen in (A). A blue arrow is added as a guide to the eye to show the shift of the absorption peak as more phonon modes are activated. Using the absorption spectra of each individual phonon mode, the contribution of each phonon mode is given through the spectral weight of the absorption spectrum concerning the regions (C) from 10 to 16 μm and (D) from 17 to 19 μm.

phase reversal is typically required[29–31] for coherent spin precession to uniquely determine the final magnetization state, these observations indicate that spin dynamics alone do not set the state selection. The polarization-dependent lattice distortion, whose sign is set by the pump polarization, is an alternative scenario. Such a scenario has been recently reported for piezomagnetic CoF_2[32] and is consistent with the piezomagnetism of $DyFeO_3$ in the AFM phase.[33] Verifying this mechanism directly would require mode-resolved lattice-sensitive probes, which are beyond the scope of the present study. Therefore, here we employ the pump-polarization control of the phonon-driven magnetization as an indicator to distinguish between non-thermal and thermal regimes and to identify their crossover timescale.

To assess the degree of control, we compute the intensity deviation, defined as $\sigma_I = \sqrt{\frac{\sum_i I(\theta_i)^2}{N}}$, where $I(\theta_i)$ is the integrated MO signal for each polarization angle θ_i and N the total number of angles measured. The value of σ_I quantifies the extent to which the MIR-induced magnetic domain distribution deviates from a zero-net-magnetization state, thereby serving as a measure of magnetization control. The inset in Figure 4B shows a suppression of σ_I over time, reflecting the gradual loss of polarization control. We approximate this temporal dependence of $\sigma_I(t)$ by a linear function as a first-order description, since the leading term of an exponential relaxation of the non-thermal component is linear in time (Figure 3C). Linear extrapolation of $\sigma_I(t)$ to zero yields a crossover time of 480 ps. This timescale is of the same order of magnitude as $\tau_{non\text{-}th}$, supporting the idea of the non-thermal-to-thermal crossover in the WFM phase of $DyFeO_3$.

Characterizing the phonon absorption spectrum

Finally, to investigate the relationship between the MIR-induced magnetization and the phonon modes, we return to the macropulse excitation scheme. We keep the pump fluence constant at F = 91 mJ/cm^2 and continuously vary the central wavelength of the pump macropulse λ from 10 to 19 μm. The efficiency of this process is evaluated by measuring the diameter, d, of the switched region. In this range of the pump wavelength, d exhibits strong sensitivity to λ and shows two distinct bands with maxima at $\lambda_1^{max} \approx 14$ μm and $\lambda_2^{max} \approx 18.5$ μm (Figure 5A). In addition, the fluence threshold F_c required to induce the PT is also dependent on λ (Note S3). Notably, the value of 1/F_c, serving as a measure of the pump-wavelength-dependent efficiency for driving the phase transition, is significantly enhanced when the pump wavelength aligns with λ^{max} (Figure 5A).

The dependence of both d and 1/F_c follows the phonon absorption spectrum $A(\lambda)$, highlighted by the orange shaded region in Figure 5A, suggesting that phonon absorption is the primary driver of the MIR-induced magnetization. To attribute these absorptions to specific phonon modes, we extracted the complex dielectric permittivity function $\epsilon(\lambda)$ by simultaneous fitting of the reflectivity $R(\lambda)$ and transmission $T(\lambda)$ spectra to a multi-oscillator

Drude-Lorentz model (Note S2). The resulting model accurately reproduces the absorption spectrum (Figure S2C). From the Drude-Lorentz model, we extract the resonance wavelengths for both the transverse optical (TO) and the longitudinal optical (LO) phonon modes, depicted as black and red dashed lines throughout Figure 5A. Two TO phonon modes can be identified here, at λ_1^{TO} = 17.9 μm and λ_2^{TO} = 19.4 μm alongside two corresponding LO phonon modes at λ_1^{LO} = 15.3 μm and λ_2^{LO} = 18.6 μm. While their wavelength matches the phonon modes predicted by density functional theory (DFT) calculations,[20,34] neither λ^{TO} nor λ^{LO} aligns with the phonon absorption and, thus, λ^{max}. This discrepancy is particularly striking for the shortest-wavelength absorption band centered at $\lambda \approx 14.5$ μm, which lies entirely outside of the associated Reststrahlen band, defined by the range between λ_1^{LO} and λ_1^{TO} (see Figure 1B). To understand the origin of this discrepancy, we examine the contribution of each phonon mode to the absorption spectrum $A(\lambda)$. Figure 5B shows a sequence of $A(\lambda)$ spectra, where phonon mode contributions are calculated and added cumulatively, starting from the mode with the longest resonance wavelength λ_9^{TO} = 53 μm. Depending on the mode strength, each phonon can produce significant absorption "wings" that extend micrometers beyond its TO resonance wavelength (Note S6). These wings carry significant spectral weight and thus can collectively shift the absorption spectrum away from the original TO resonances, thereby explaining the blue shift of λ^{max} relative to the nearby λ^{TO}. Remarkably, the observed magnetic response occurs at significantly lower excitation wavelengths than those associated with LO phonons, which have been widely proposed as a mechanism for various phonon-driven phase transitions.[18,35,36] This indicates that LO phonons likely do not play a dominant role in the Morin phase transition under our experimental conditions, since their direct excitation is suppressed for normal-incidence optical pumping.

Additionally, to estimate the contribution of each phonon mode to the absorption bands, we activate each individual mode within the Drude-Lorentz model one by one and calculate their absorption spectra (Figure S5). Using these absorption spectra, we evaluate the spectral weight—and thus the contribution—of each phonon mode within two spectral windows: 10–16 μm (Figure 5C) and 17–19 μm (Figure 5D). These spectral windows correspond to the regions where the MIR-induced magnetization was observed. One can see that while the B_{1u} mode at λ_1^{TO} = 17.9 μm is the primary contributor to the absorption in the 10- to 16-μm window, other phonon modes also contribute significantly. In contrast, the contribution from other modes is less pronounced in the 17- to 19-μm range, where the absorption is dominated by two closely spaced phonon modes at λ_1^{TO} and λ_2^{TO}. Although the second absorption peak is situated closer to λ_2^{TO}, its absorption is dominated by the λ_1 phonon. This suggests that pumping either resonance has a similar impact on the magnetic state of $DyFeO_3$. Indeed, we observe that micropulse excitation not only reproduces the wavelength dependence of the MIR-induced magnetization seen with macropulses (Figure S10) but also that pumping at λ_2^{max} = 18.5 μm induces a net magnetization with dynamics closely resembling those driven by excitation at λ_1^{max} = 14 μm (Figure S11). The primary contribution toward both absorption windows is the B_{1u} phonon mode, previously predicted through DFT calculations to non-linearly couple to a Raman-active A_g phonon mode, as stated in Afanasiev et al.[20]

DISCUSSION

Our experiments reveal two temporal regimes in the phonon-induced ultrafast AFM-to-WFM phase transition in $DyFeO_3$, clearly distinguished by signatures in their spatial profile and pump-polarization dependences. The MIR-induced dynamics begin with an initial non-thermal response that is spatially homogeneous and sensitive to pump polarization, followed by a thermal multidomain state that is insensitive to the pump polarization. We find that the crossover between these regimes occurs on the timescale of about 200 ps, closely matching the spin-lattice relaxation time in the WFM phase. Although our MO experiments demonstrate a thermalization pathway of the magnetic system, magneto-optics remain largely insensitive to the state of the underlying lattice. To further clarify the role of the lattice in this process, time-resolved X-ray studies will be essential to determine whether the lattice remains dynamically coupled to the spin system or relaxes on the timescale of the excited phonon coherence. Crucially, we identify resonant phonon absorption as the primary driver of the phase transition, and by applying a multi-oscillator Lorentz model, we show that the relevant absorption bands are dominated by the same high-frequency IR-active phonon modes. Our results therefore demonstrate that systematic, wavelength-resolved studies spanning multiple phonon resonances as well as multiple timescales are essential for disentangling the microscopic origin of ultrafast phonon-induced phase transitions and for correctly interpreting mode-selective control in complex magnetic materials.

METHODS

$DyFeO_3$ single crystal

The monocrystalline $DyFeO_3$ sample used is grown by floating-zone melting. It is 63 μm thick and the dimensions are 3.5 × 3.5 mm across, cut along the crystallographic *c* axis. To control the temperature, we placed the $DyFeO_3$ sample in an optical cryostat from where liquid helium was pumped into the system, allowing for temperatures down to 5 K.

MO pump-probe microscopy experiments

The excitation and detection of the PT dynamics of the sample were carried out using MIR pump light pulses generated by the free-electron laser facility FELIX in Nijmegen, the Netherlands. The micropulses within the macropulse have a wavelength-dependent duration of 1–3 ps, with tunable bandwidths ranging from 0.5% to 2% and central wavelengths adjustable between λ = 3 μm and λ = 100 μm.[27]

Unless otherwise specified, the macropulse energy at the sample position was measured to be about 40 μJ. The micropulse energy was measured to be 2.4 μJ. The micropulses are focused onto the sample surface using a 90° off-axis parabolic mirror, forming an elliptical spot with full-width-at-half-maximum dimensions of 300 × 130 μm, as determined by the Liu method.[37,38] To perform MO imaging, we probe the sample

CellPress
OPEN ACCESS

with linearly polarized light obtained from either a CW He:Ne laser with a central wavelength of 633 nm when using the macropulse scheme or a regeneratively amplified Ti:sapphire pulsed laser with a central wavelength of 800 nm and a pulse duration of 25 fs during the micropulse experiments. This transmitted probe light is collected by an objective lens, filtered by an analyzer, and captured by a CCD camera. The polarization rotation induced by the MO Faraday effect allows us to directly resolve the spatial distribution of magnetization across the sample and detect pump-induced switching between the AFM and WFM phases.

By electronically gating the delay between the arrival of the macropulse and the 27-μs-long camera exposure, we can capture both the dynamics occurring during the macropulse exposure and the subsequent relaxation processes evolving over microsecond-to-millisecond time scales. To resolve the ultrafast dynamics, we use single micropulses and perform MO imaging using the 25-fs pulses from the Ti:sapphire laser to illuminate the sample.

FTIR reflectivity spectrum

Transmission and reflection spectra were measured with a Bruker Vertex 80v FTIR spectrometer. In both measurement modes, the IR beam was focused to a diameter of approximately 1 mm at the sample position to accommodate the small sample size. Care was taken to ensure that only the sample was illuminated. Reflection measurements were performed using the commercial Bruker reflection module A517, which features a fixed angle of incidence of 30°. Measurements were made relative to an uncoated gold reference surface.

The reduced beam diameter, and consequently lower signal levels, made the setup more susceptible to spurious IR contributions from various components of the spectrometer (e.g., source and detector) and from the sample environment. These parasitic signals effectively broaden the IR spot size and introduce a phase error in the signal. A four-scan measurement approach similar to that proposed by Kehrt et al.[39] was employed to suppress unwanted background contributions.

The modeling performed was carried out using the data-analysis program RefFIT by Kuzmenko.[40] Further details about fitting the FTIR data are available in Note S2. Finding the TO and LO phonon modes was accomplished in two ways. The TO modes can be found as the direct parameter ω_T (the resonance frequency of the oscillator) in the performed model in Table S1. Through the energy loss function, $Im\left(\frac{-1}{\tilde{\epsilon}(\omega)}\right)$, one can identify the resonance frequencies of LO phonon modes by finding the maxima within the function.

Phonon calculations through DFT

Determination and representations of the phonon modes were carried out using DFT calculations published in Afanasiev et al.[20,41] and Gareev et al.[20,41] The *Pnma* phase of $DyFeO_3$ and its projected augmented wave were simulated using the ABINIT package.[42] The phonon calculation was performed through the frozen phonon technique with phonopy software.[43]

RESOURCE AVAILABILITY

Lead contact

Requests for further information and resources should be directed to and will be fulfilled by the lead contact, Jim Groefsema (jim.groefsema@ru.nl).

Materials availability

This study did not generate new unique materials.

Data and code availability

- All data and original code have been deposited at the Radboud Data Repository and are publicly available at https://doi.org/10.34973/1m7a-kg25 as of the date of publication.
- Any additional information required to reanalyze the data reported in this paper is available from the lead contact upon request.

ACKNOWLEDGMENTS

We are grateful to K. Saeedi and C. Berkhout for technical support, A. Sasani for conducting DFT calculations to define phonon modes and fruitful discussions, A. Dolgikh for supplying the initial render of the pump-probe setup, E. Bousquet for his additional assistance on the DFT calculations, and B.A. Ivanov for fruitful discussions on the results. We acknowledge funding from ERC grant 101078206 ASTRAL; ERC grant 101115234 HANDSHAKE; program “Materials for the Quantum Age” (QuMat, registration number 024.005.006), which is part of the Gravitation program financed by the Dutch Ministry of Education, Culture and Science (OCW); the European Research Council ERC grant agreement no. 101054664 (SPARTACUS) and grant agreement no. 856538 (3D-MAGiC); and the European Union’s Horizon 2020 Research and Innovation Program under Marie Skłodowska-Curie grant agreement no. 861300 (COMRAD). M.X.N. acknowledges support from the Natural Sciences and Engineering Research Council of Canada (NSERC) PDF fellowship. We acknowledge support from the Netherlands Initiative for Energy-Efficient Computing (NL-ECO), part of the Dutch National Science Agenda (NWA-ORC), grant agreement no. NWA.1389.20.140.

AUTHOR CONTRIBUTIONS

Conceptualization, C.S.D., A.I.K., A.D.C., A.V.K., and D.A.; funding acquisition, C.S.D., A.I.K., A.V.K., T.H.M.R., and D.A.; resources, C.S.D., A.I.K., A.V.K., and D.A.; investigation, J.G., V.R., T.J., N.D., V.B., C.S.D., A.V.K., and D.A.; analysis, J.G., V.R., P.K.K., T.T.G., and D.A.; visualization, J.G., V.R., P.K.K., M.X.N., and D.A.; writing – original draft, J.G., P.K.K., A.V.K., and D.A.; writing – review & editing, V.R., T.J., M.X.N., T.T.G., J.R.H., T.H.M.R., A.I.K., and C.S.D.; supervision, D.A.

DECLARATION OF INTERESTS

The authors declare no competing interests.

DECLARATION OF GENERATIVE AI AND AI-ASSISTED TECHNOLOGIES IN THE WRITING PROCESS

No generative AI tools were used during the preparation of this work.

SUPPLEMENTAL INFORMATION

Supplemental information can be found online at https://doi.org/10.1016/j.newton.2026.100509.

Received: August 7, 2025
Revised: December 22, 2025
Accepted: April 1, 2026
Published: April 24, 2026

REFERENCES

1. Mankowsky, R., von Hoegen, A., Först, M., and Cavalleri, A. (2017). Ultrafast Reversal of the Ferroelectric Polarization. Phys. Rev. Lett. *118*, 197601. https://doi.org/10.1103/PhysRevLett.118.197601.
2. Henstridge, M., Först, M., Rowe, E., Fechner, M., and Cavalleri, A. (2022). Nonlocal nonlinear phononics. Nat. Phys. *18*, 457–461. https://doi.org/10.1038/s41567-022-01512-3.
3. Afanasiev, D., Ivanov, B.A., Kirilyuk, A., Rasing, T., Pisarev, R.V., and Kimel, A.V. (2016). Control of the Ultrafast Photoinduced Magnetization across the Morin Transition in $DyFeO_3$. Phys. Rev. Lett. *116*, 097401. https://doi.org/10.1103/PhysRevLett.116.097401.
4. Disa, A.S., Nova, T.F., and Cavalleri, A. (2021). Engineering crystal structures with light. Nat. Phys. *17*, 1087–1092. https://doi.org/10.1038/s41567-021-01366-1.
5. Nova, T.F., Disa, A.S., Fechner, M., and Cavalleri, A. (2019). Metastable ferroelectricity in optically strained $SrTiO_3$. Science *364*, 1075–1079. https://doi.org/10.1126/science.aaw4911.
6. Gattinoni, C., Strkalj, N., Härdi, R., Fiebig, M., Trassin, M., and Spaldin, N.A. (2020). Interface and surface stabilization of the polarization in ferroelectric thin films. Proc. Natl. Acad. Sci. USA *117*, 28589–28595. https://doi.org/10.1073/pnas.2007736117.
7. Fausti, D., Tobey, R.I., Dean, N., Kaiser, S., Dienst, A., Hoffmann, M.C., Pyon, S., Takayama, T., Takagi, H., and Cavalleri, A. (2011). Light-Induced Superconductivity in a Stripe-Ordered Cuprate. Science *331*, 189–191. https://doi.org/10.1126/science.1197294.
8. Cavalleri, A., Dekorsy, T., Chong, H.H.W., Kieffer, J.C., and Schoenlein, R.W. (2004). Evidence for a structurally-driven insulator-to-metal transition in VO_2: A view from the ultrafast timescale. Phys. Rev. B *70*, 161102. https://doi.org/10.1103/PhysRevB.70.161102.
9. Caviglia, A.D., Scherwitzl, R., Popovich, P., Hu, W., Bromberger, H., Singla, R., Mitrano, M., Hoffmann, M.C., Kaiser, S., Zubko, P., et al. (2012). Ultrafast Strain Engineering in Complex Oxide Heterostructures. Phys. Rev. Lett. *108*, 136801. https://doi.org/10.1103/PhysRevLett.108.136801.
10. Buzzi, M., Nicoletti, D., Fechner, M., Tancogne-Dejean, N., Sentef, M.A., Georges, A., Biesner, T., Uykur, E., Dressel, M., Henderson, A., et al. (2020). Photomolecular High-Temperature Superconductivity. Phys. Rev. X *10*, 031028. https://doi.org/10.1103/PhysRevX.10.031028.
11. Juraschek, D.M., Fechner, M., Balatsky, A.V., and Spaldin, N.A. (2017). Dynamical multiferroicity. Phys. Rev. Mater. *1*, 014401. https://doi.org/10.1103/PhysRevMaterials.1.014401.
12. Juraschek, D.M., Neuman, T., and Narang, P. (2022). Giant effective magnetic fields from optically driven chiral phonons in *4f* paramagnets. Phys. Rev. Res. *4*, 013129. https://doi.org/10.1103/PhysRevResearch.4.013129.
13. Romao, C.P., and Juraschek, D.M. (2024). Light makes atoms behave like electromagnetic coils. Nature *628*, 505–506. https://doi.org/10.1038/d41586-024-00889-w.
14. Basini, M., Pancaldi, M., Wehinger, B., Udina, M., Unikandanunni, V., Tadano, T., Hoffmann, M.C., Balatsky, A.V., and Bonetti, S. (2024). Terahertz electric-field-driven dynamical multiferroicity in SrTiO3. Nature *628*, 534–539. https://doi.org/10.1038/s41586-024-07175-9.
15. de la Torre, A., Kennes, D.M., Claassen, M., Gerber, S., McIver, J.W., and Sentef, M.A. (2021). Colloquium: Nonthermal pathways to ultrafast control in quantum materials. Rev. Mod. Phys. *93*, 041002. https://doi.org/10.1103/RevModPhys.93.041002.
16. Maehrlein, S.F., Radu, I., Maldonado, P., Paarmann, A., Gensch, M., Kalashnikova, A.M., Pisarev, R.V., Wolf, M., Oppeneer, P.M., Barker, J., and Kampfrath, T. (2018). Dissecting spin-phonon equilibration in ferrimagnetic insulators by ultrafast lattice excitation. Sci. Adv. *4*, eaar5164. https://doi.org/10.1126/sciadv.aar5164.
17. Matthiesen, M., Hortensius, J.R., Mañas-Valero, S., Kapon, I., Dumcenco, D., Giannini, E., Šiškins, M., Ivanov, B.A., van der Zant, H.S.J., Coronado, E., et al. (2023). Controlling Magnetism with Light in a Zero Orbital Angular Momentum Antiferromagnet. Phys. Rev. Lett. *130*, 076702. https://doi.org/10.1103/PhysRevLett.130.076702.
18. Stupakiewicz, A., Davies, C.S., Szerenos, K., Afanasiev, D., Rabinovich, K.S., Boris, A.V., Caviglia, A., Kimel, A.V., and Kirilyuk, A. (2021). Ultrafast phononic switching of magnetization. Nat. Phys. *17*, 489–492. https://doi.org/10.1038/s41567-020-01124-9.
19. Davies, C.S., Fennema, F.G.N., Tsukamoto, A., Razdolski, I., Kimel, A.V., and Kirilyuk, A. (2024). Phononic switching of magnetization by the ultrafast Barnett effect. Nature *628*, 540–544. https://doi.org/10.1038/s41586-024-07200-x.
20. Afanasiev, D., Hortensius, J.R., Ivanov, B.A., Sasani, A., Bousquet, E., Blanter, Y.M., Mikhaylovskiy, R.V., Kimel, A.V., and Caviglia, A.D. (2021). Ultrafast control of magnetic interactions via light-driven phonons. Nat. Mater. *20*, 607–611. https://doi.org/10.1038/s41563-021-00922-7.
21. Cui, R., Jiang, H., Du, Y., Xu, Y., Jia, Y., Sun, K., and Hao, X. (2024). Role of Dy *4f* electrons on magnetic coupling and reorientation in $DyFeO_3$. J. Phys. Condens. Matter *36*, 335501. https://doi.org/10.1088/1361-648X/ad49f9.
22. Hoogeboom, G.R., Kuschel, T., Bauer, G.E.W., Mostovoy, M.V., Kimel, A.V., and van Wees, B.J. (2021). Magnetic order of Dy^{3+} and Fe^{3+} moments in antiferromagnetic $DyFeO_3$ probed by spin Hall magnetoresistance and spin Seebeck effect. Phys. Rev. B *103*, 134406. https://doi.org/10.1103/PhysRevB.103.134406.
23. Li, E., Feng, Z., Kang, B., Zhang, J., Ren, W., and Cao, S. (2019). Spin switching in single crystal PrFeO3 and spin configuration diagram of rare earth orthoferrites. J. Alloys Compd. *811*, 152043. https://doi.org/10.1016/j.jallcom.2019.152043.
24. Prelorendjo, L.A., Johnson, C.E., Thomas, M.F., and Wanklyn, B.M. (1980). Spin reorientation transitions in $DyFeO_3$ induced by magnetic fields. J. Phys. C Solid State Phys. *13*, 2567–2578. https://doi.org/10.1088/0022-3719/13/13/012.
25. Bazaliy, Y.B., Tsymbal, L.T., Kakazei, G.N., Izotov, A.I., and Wigen, P.E. (2004). Spin-reorientation in $ErFeO_3$: Zero-field transitions, three-dimensional phase diagram, and anisotropy of erbium magnetism. Phys. Rev. B *69*, 104429. https://doi.org/10.1103/PhysRevB.69.104429.
26. Jamil, M.T., Ahmad, J., Bukhari, S.H., and Ahmad, H. (2018). Optical phonons and its effect on physical properties of rare-earth orthoferrites $RFeO_3$ (R = La, Nd, Gd, Dy, Er): IR reflectivity measurements. Int. J. Mod. Phys. B *32*, 1850229. https://doi.org/10.1142/S0217979218502296.
27. Knippels, G.M.H., and van der Meer, A.F.G. (1998). FEL diagnostics and user control. Nucl. Instrum. Methods Phys. Res. Sect. B Beam Interact. Mater. Atoms *144*, 32–39. https://doi.org/10.1016/S0168-583X(98)00347-4.
28. Yamaguchi, K., Kurihara, T., Watanabe, H., Nakajima, M., and Suemoto, T. (2015). Dynamics of photoinduced change of magnetoanisotropy parameter in orthoferrites probed with terahertz excited coherent spin precession. Phys. Rev. B *92*, 064404. https://doi.org/10.1103/PhysRevB.92.064404.
29. Khokhlov, N.E., Dolgikh, A.E., Ivanov, B.A., and Kimel, A.V. (2024). Double pulse all-optical coherent control of ultrafast spin-reorientation in an antiferromagnetic rare-earth orthoferrite. APL Mater. *12*, 051119. https://doi.org/10.1063/5.0197976.
30. de Jong, J.A., Razdolski, I., Kalashnikova, A.M., Pisarev, R.V., Balbashov, A.M., Kirilyuk, A., Rasing, T., and Kimel, A.V. (2012). Coherent Control of the Route of an Ultrafast Magnetic Phase Transition via Low-Amplitude Spin Precession. Phys. Rev. Lett. *108*, 157601. https://doi.org/10.1103/PhysRevLett.108.157601.
31. Kimel, A.V., Ivanov, B.A., Pisarev, R.V., Usachev, P.A., Kirilyuk, A., and Rasing, T. (2009). Inertia-driven spin switching in antiferromagnets. Nat. Phys. *5*, 727–731. https://doi.org/10.1038/nphys1369.
32. Disa, A.S., Fechner, M., Nova, T.F., Liu, B., Först, M., Prabhakaran, D., Radaelli, P.G., and Cavalleri, A. (2020). Polarizing an antiferromagnet by

CellPress
OPEN ACCESS

optical engineering of the crystal field. Nat. Phys. *16*, 937–941. https://doi.org/10.1038/s41567-020-0936-3.

33. Eremenko, V.V., and Kharchenko, N.F. (1987). Magneto-optics of antiferromagnets. Phys. Rep. *155*, 379–401. https://doi.org/10.1016/0370-1573(89)90066-5.
34. Gupta, H.C., Kumar Singh, M., and Tiwari, L.M. (2002). Lattice dynamic investigation of Raman and infrared wavenumbers at the zone center of orthorhombic $RFeO_3$ (R = Tb, Dy, Ho, Er, Tm) perovskites. J. Raman Spectrosc. *33*, 67–70. https://doi.org/10.1002/jrs.805.
35. Kwaaitaal, M., Lourens, D.G., Davies, C.S., and Kirilyuk, A. (2024). Epsilon-near-zero regime enables permanent ultrafast all-optical reversal of ferroelectric polarization. Nat. Photon. *18*, 569–573. https://doi.org/10.1038/s41566-024-01420-3.
36. Davies, C.S., and Kirilyuk, A. (2024). Epsilon-near-zero regime for ultrafast opto-spintronics. Npj Spintron. *2*, 20–26. https://doi.org/10.1038/s44306-024-00025-4.
37. Gidding, M., Janssen, T., Davies, C.S., and Kirilyuk, A. (2023). Dynamic self-organisation and pattern formation by magnon-polarons. Nat. Commun. *14*, 2208. https://doi.org/10.1038/s41467-023-37919-6.
38. Liu, J.M. (1982). Simple technique for measurements of pulsed Gaussian-beam spot sizes. Opt. Lett. *7*, 196–198. https://doi.org/10.1364/OL.7.000196.
39. Kehrt, M., Monte, C., Steiger, A., and Hollandt, J. (2018). Background corrected measurements of optical quantities in the far-infrared spectral range. Opt. Express *26*, 34002–34006. https://doi.org/10.1364/OE.26.034002.
40. Kuzmenko, A.B. (2005). Kramers–Kronig constrained variational analysis of optical spectra. Rev. Sci. Instrum. *76*, 083108. https://doi.org/10.1063/1.1979470.
41. Gareev, T.T., Sasani, A., Khusyainov, D.I., Bousquet, E., Gareeva, Z.V., Kimel, A.V., and Afanasiev, D. (2024). Optical Excitation of Coherent THz Dynamics of the Rare-Earth Lattice through Resonant Pumping of $f-f$ Electronic Transition in a Complex Perovskite DyFeO3. Phys. Rev. Lett. *133*, 246901. https://doi.org/10.1103/PhysRevLett.133.246901.
42. Gonze, X., Jollet, F., Abreu Araujo, F., Adams, D., Amadon, B., Applencourt, T., Audouze, C., Beuken, J.-M., Bieder, J., Bokhanchuk, A., et al. (2016). Recent developments in the ABINIT software package. Comput. Phys. Commun. *205*, 106–131. https://doi.org/10.1016/j.cpc.2016.04.003.
43. Togo, A., and Tanaka, I. (2015). First principles phonon calculations in materials science. Scr. Mater. *108*, 1–5. https://doi.org/10.1016/j.scriptamat.2015.07.021.

NEWTON, Volume *2*

Supplemental information

Emergence of two distinct regimes in phonon-induced non-equilibrium magnetization dynamics

Jim Groefsema, Viktoriia Radovskaia, Thom Janssen, Nils Dessmann, Vladislav Bilyk, Peter K. Kim, Timur T. Gareev, Meng Xing Na, Jorrit R. Hortensius, Andrea D. Caviglia, Theo H.M. Rasing, Andrei I. Kirilyuk, Carl S. Davies, Alexey V. Kimel, and Dmytro Afanasiev

Note S1. The crystal structure of $DyFeO_3$

In Fig. S1 we present the crystal structure of $DyFeO_3$ using the space group *Pnma*. The structure is of an orthorhombically distorted perovskite. The unit cell consists out of 4 formula units, where octahedrons of oxygen atoms encase the iron atoms [1,2].

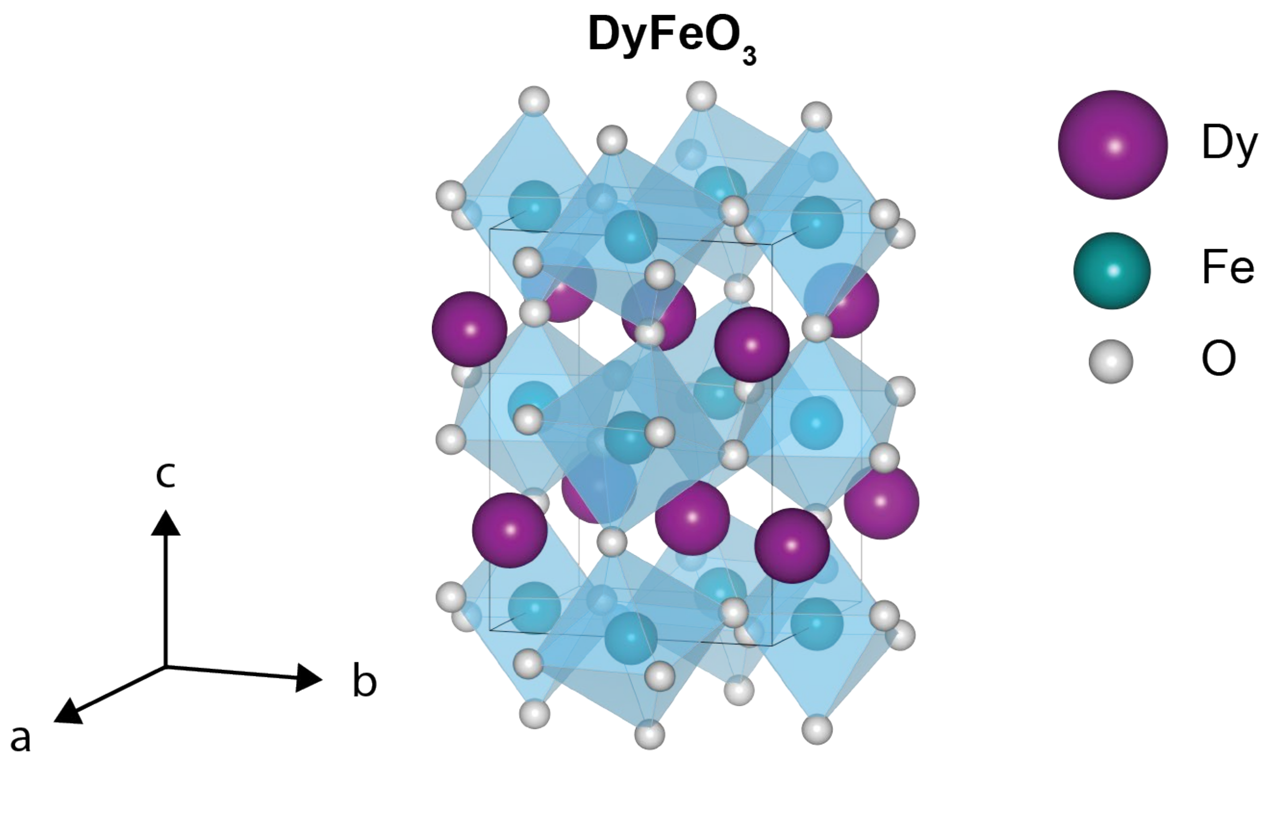

Figure S1 | Crystal structure of $DyFeO_3$. The crystal structure of $DyFeO_3$. The purple, green and white sphere represent dysprosium, iron and oxygen atoms respectively. The oxygen octahedra are shaded in blue.

Note S2. Fitting the FTIR spectrum

The full reflectivity spectrum of $DyFeO_3$ obtained through Fourier-transform infrared spectroscopy (FTIR) can be found in Fig. S2.

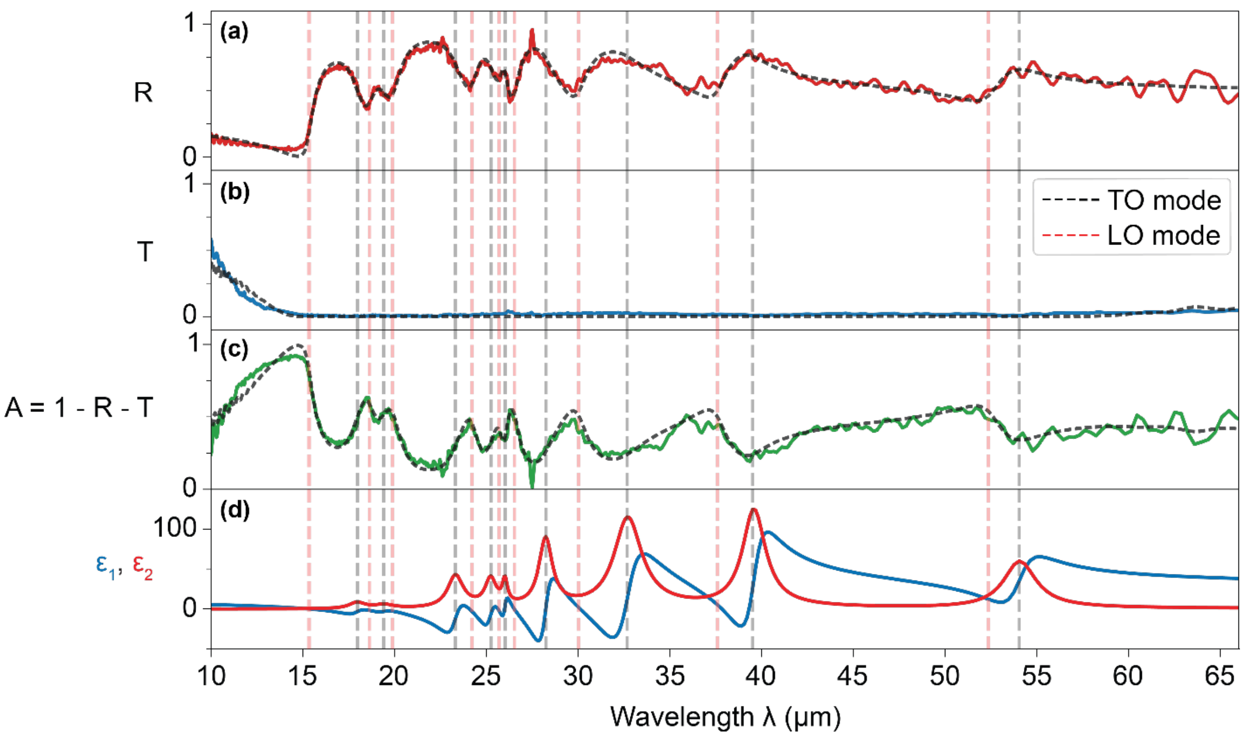

Figure S2 | The reflectivity spectrum of $DyFeO_3$. The infrared reflectivity **(a)** and transmission **(b)** spectrum of $DyFeO_3$ through Fourier-transform infrared spectroscopy (FTIR) obtained at room temperature. The absorption **(c)** is obtained through the relation $A = 1 - R - T$. The black dashed lines are the resulting spectrum obtained after fitting the data using the Drude-Lorentz model. The FTIR measurement was taken using unpolarized light. The angle of incidence used is 30°. **(d)** The calculated elements of the complex dielectric permittivity $\tilde{\epsilon}(\omega) = \epsilon_1(\omega) + i\epsilon_2(\omega)$ where $\epsilon_1(\omega)$ and $\epsilon_2(\omega)$ are the real and imaginary parts of $\tilde{\epsilon}(\omega)$ respectively.

We obtained the complex dielectric permittivity, $\tilde{\epsilon}(\omega)$ by simultaneously fitting the reflectivity and transmission spectrum to Drude-Lorentz oscillators (Eq. S2.1) and the Fresnel equations. The light source used was unpolarized and at an angle of incidence of 30°, so to describe the reflectivity and transmission data, we can take the average between *p*- and *s*-polarized light, detailed in equations S2.2 and S2.3:

$$\tilde{\epsilon}(\omega) = \epsilon_1(\omega) + i\epsilon_2(\omega) = \epsilon_\infty + \sum \frac{S_i\omega_{Tj}^2}{\omega_{Tj}^2 - \omega^2 - i\omega\gamma_j}, \qquad (S2.1)$$

$$\langle R\rangle = |\langle r\rangle|^2 = \left|\frac{r_p + r_s}{2}\right|^2 = \left|\frac{\sin^2\alpha\,(\tilde{\epsilon}(\omega) - 1)}{\left(\cos\alpha + \sqrt{\tilde{\epsilon}(\omega) - \sin^2\alpha}\right)\left(\tilde{\epsilon}(\omega)\cos\alpha + \sqrt{\tilde{\epsilon}(\omega) - \sin^2\alpha}\right)}\right|^2 \qquad (S2.2)$$

$$\langle T\rangle = \left|(1 - \langle r\rangle^2)\, e^{i\frac{\omega}{c}\sqrt{\tilde{\epsilon}(\omega)}d}\right|^2 \qquad (S2.3)$$

where ϵ_∞ is the high-frequency value of the dielectric permittivity, $\epsilon_1(\omega)$ and $\epsilon_2(\omega)$ are the real and imaginary parts of the dielectric permittivity respectively, ω_{Tj}, γ_j, and S_j are the frequency, damping, and oscillator strength of a given transverse optical (TO) phonon mode, c

is the speed of light, d the sample thickness, and r_p and r_s are the reflectance coefficients for p- and s-polarized light at an angle of incidence α. ϵ_∞ is obtained by fitting the model up to 7000 cm^{-1}. To see how well this fitting procedure follows the reflectivity spectrum, Fig. S2A and B shows both the experimentally found data points and the resulting fit using Eq. S2.1. Fig. S2C shows the absorption spectrum for both the data points and the resulting fit obtained through the relation $A = 1 - R - T$, where A is the absorption, R the reflectivity and T the transmission. Fig. S2D shows the resulting dielectric permittivity, decomposed into the real part ϵ_1 and the imaginary part ϵ_2 as obtained through the Drude-Lorentz fitting procedure.

Nine Drude-Lorentz oscillators were identified and fitted. The parameters used for the fitting procedure can be found in table S1. Using the connection $\omega = \frac{2\pi c}{\lambda}$, where c is the speed of light and λ the wavelength, we can write ω_T and ω as λ_T and λ respectively. $\epsilon_\infty$was found to be 7.8 by fitting the high frequency part of the FTIR data. We compared the results to the phonon mode frequencies reported in Ref [3], but since we used unpolarized light to capture the reflectivity spectrum, the phonon symmetry mode cannot be accurately determined. As a result, the phonon modes in table S1 are only a suggestion for the potential mode that is resonant at this wavelength.

**Phonon mode $\lambda_\#$**	**λ_T (µm)**	**S**	**Y (µm)**	**Suggested phonon mode**	**DFT λ_T (µm)**
9	54.06	2.32	1371.55	B_{3u}(2)	53.49
8	39.58	4.67	1036.01	B_{1u}(3)	39.99
7	32.70	6.34	582.36	B_{3u}(5)	32.36
6	28.23	2.67	902.54	B_{2u}(5)	28.49
5	26.01	0.36	2100.79	-	-
4	25.23	1.00	861.75	B_{1u}(7)	25.45
3	23.31	1.81	522.69	B_{3u} (7)	23.58
2	19.44	0.28	321.13	B_{3u} (8)	19.45
1	17.96	0.47	300.45	B_{1u} (9)	18.42

Table S1 | $DyFeO_3$ reflectivity fitting parameters. The calculated parameters of the transverse optical (TO) phonon modes. The parameters are λ_T, S and γ, which represent the wavelength, oscillator strength and damping of a given TO phonon mode. Nine modes have been found and described. A suggestion of the potential phonon mode along with its calculated wavelength is given using the DFT calculations as reported in Ref. [3].

Figure S3 shows the schematic of every modelled phonon eigenmode as found in Table S1. The lattice distortions correspond to a stretching of the oxygen bonds, displayed as yellow arrows.

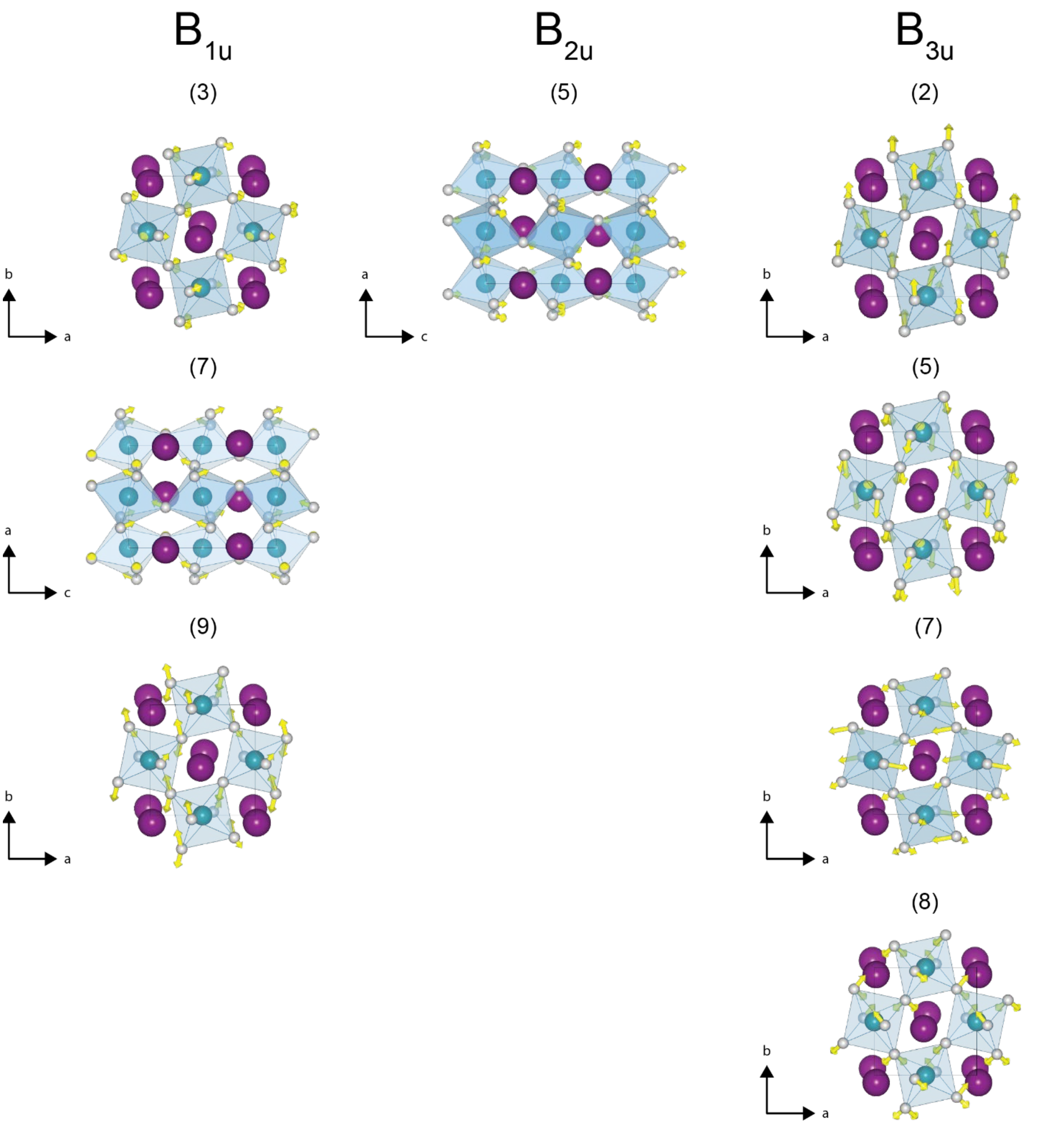

Figure S3 | Calculated phonon eigenmodes. Eight phonon eigenmodes are displayed here. Three corresponding to the B_{1u} mode, one to the B_{2u} mode and four to the B_{3u} mode. The purple/blue/gray spheres correspond to dysprosium, iron and oxygen atoms. The oxygen octahedra are shaded in light blue. Yellow arrows show the movement caused by the phonon eigenmode. The bracketed numbers correspond to the degeneracy as found in Ref [3].

Note S3. Fluence dependence of the macropulse measurement at different wavelengths

We measured the fluence dependence to determine the critical fluence above which the PT is triggered. To obtain a threshold fluence F_c at which a phonon-induced transition occurs, we first assume a Gaussian fluence distribution for our laser pulse,

$$F = F_0\, e^{\frac{-x^2}{2\sigma^2}}, \qquad (S3.1)$$

where F_0 is the peak fluence, x is a spatial coordinate in the plane of the sample, and σ the beam diameter. The Gaussian profile has been illustrated in Fig. S3.1a. We can define a critical threshold fluence, F_c as the fluence at the boundary of the phonon-induced WFM state observed through MO imaging at $x_c \equiv d/2$, where d is the diameter. Thus we can write,

$$d = 2\sigma\sqrt{2\ln\left(\frac{F_0}{F_c}\right)}. \qquad (S3.2)$$

We experimentally measure the fluence dependence of the diameter of phonon-induced state at various wavelengths as shown in Fig. S3.1b. We can fit the data to Using Eq. S3.2, to extract a critical threshold fluence for each wavelength used, F_c. These fit values for $1/F_c$ are shown in Fig. 5a as stars.

Next, to fit the measured diameter data d at constant fluence F_0 and varying wavelength, as seen in Fig. 5a*,* Eq. S3.2 was used where $1/F_c$ is modeled as a sum of two Gaussian functions with two distinct maxima at the wavelengths λ_{max}=14 µm and λ_{max}=18.5 µm in accordance with our findings from the fluence dependence measurement. In this fitting procedure we have taken σ to be 354 µm as obtained through the fitting procedure of the fluence dependence and F_0 has been kept constant at 91 mJ/cm^2. The two summed Gaussian functions can be written as the following,

$$\mathrm{F_c}(\lambda) = \alpha_1 e^{-\frac{(\lambda-\mu_1)^2}{2\rho_1{}^2}} + \alpha_2 e^{-\frac{(\lambda-\mu_2)^2}{2\rho_2{}^2}}, \qquad (S3.3)$$

where $\alpha_{1,2}$ is the inverse of the threshold fluence F_c at resonance, λ is the pump wavelength, $\mu_{1,2}$ resonance wavelength and $\rho_{1,2}$ the spectral width of the F_c peaks. Considering the agreement of the fitting results using Eq. S3.2 and Eq. S3.3 with the diameter data, we can conclude that the nature of the phonon-induced PT necessitates a critical fluence to occur that is strongly wavelength dependent with two clear resonances at λ_{max}=14 µm and λ_{max}=18.5 µm.

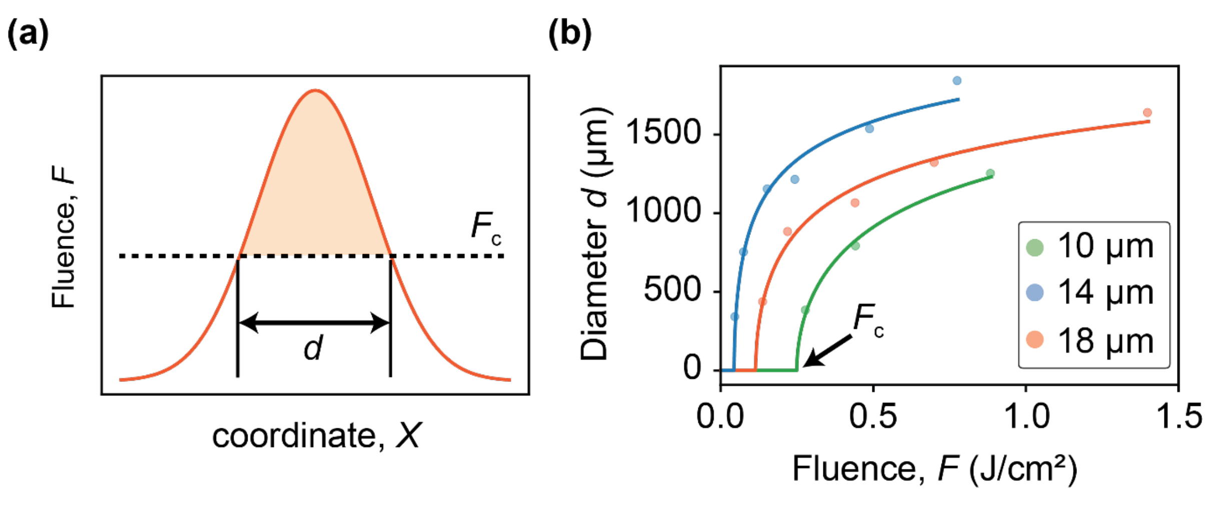

Figure S4 | Fitting the fluence dependence. (a) The spatial Gaussian distribution of the fluence of the pump pulse. The dashed line represents the critical fluence F_c above which the photo-induced phase transition occurs. The diameter d is defined here as the edge of the coordinate X where the phase transition is visible. **(b)** The diameter d of the photo-induced WFM region as a function of the mid-infrared (MIR) pump fluence in J/cm^2. The solid line is a logarithmic fit for the data points as described in Eq. (S3.2). The value at which d reaches zero is marked by an arrow and referred to as the critical fluence F_c. Three pump wavelengths are shown, marked by their own separate color.

Note S4. Phononic contributions to the absorption spectrum

To further understand the contribution of each phonon mode to the absorption spectrum, Fig. S5 presents each phonon mode in terms of their absorption using the Drude-Lorentz model and Eqs. S2.2 and S2.3. We see that depending on the strength of the oscillator, we can obtain significant absorption "wings" stretching out several micrometers beyond its resonance wavelength. A splitting occurs in the absorption due to the LO-TO splitting, creating a Reststrahlen band in between. The indices of every mode seen in Fig. S5 corresponds to the phonons with the same labels in Fig. 5 and table S1.

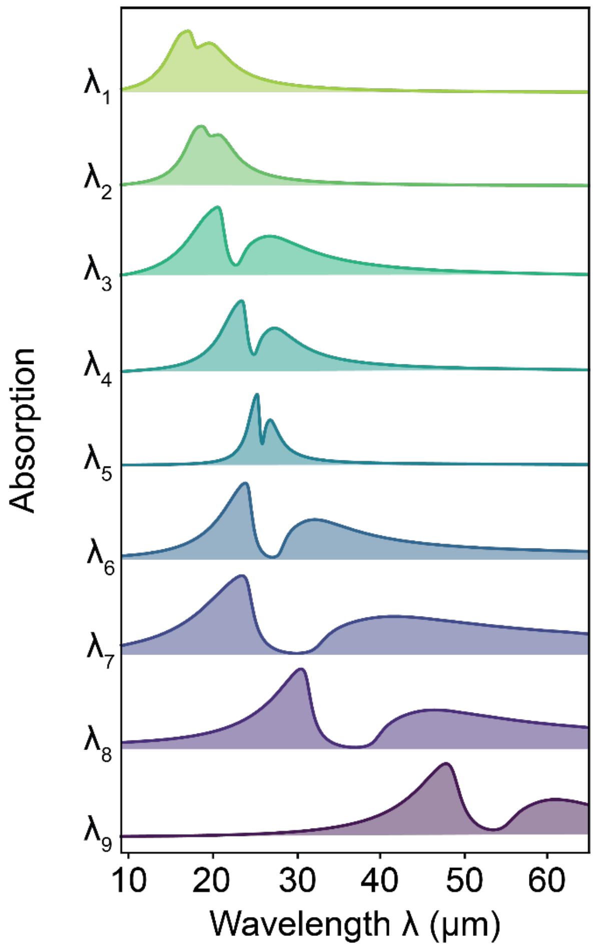

Figure S5 | Phonon contributions to the absorption model. (a) The absorption spectrum for every calculated phonon mode is shown separately with a differently colored shaded curve.

Note S5. Fluence dependence of the micropulse

Our imaging experiments reveal that the micropulse fluence dependence closely follows that observed in the macropulse regime, exhibiting a clear threshold behaviour (see Fig. S6). When the fluence exceeds this threshold, the area undergoing the phase transition increases, resulting in a larger overall magnetization change.

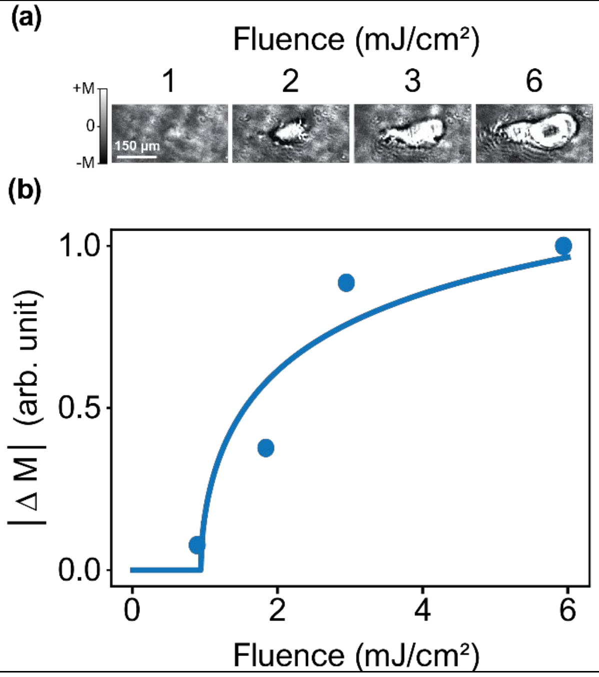

Figure S6 | Micropulse fluence dependence (a) Magneto-optical images of the phonon-induced spot using micropulses in a single-shot regime at various given fluences. **(b)** The change in magnetization as a function of the micropulse fluence. A blue curve is given as a guide to the eye.

To estimate how the lifetime of the nonthermal state evolves as a function of fluence, we present in Fig. S7 the magnetization dynamics extracted from vertical slices of the MO images at different distances from the beam center. Assuming a Gaussian excitation profile, the position of each slice provides an estimate of the local pump fluence. Using this approach, we determine the lifetime of the non-thermal process, $\tau_{non\text{-}th}$, as a function of the pump fluence, showing that the lifetime of the nonthermal, phonon-driven process is only weakly dependent on the pump fluence, see Fig. S7C. At higher pump fluences, $\tau_{non\text{-}th}$ falls slightly compared to the lower fluences, following the lifetimes of the WFM precession modes [4], known to be governed by the spin-lattice interaction.

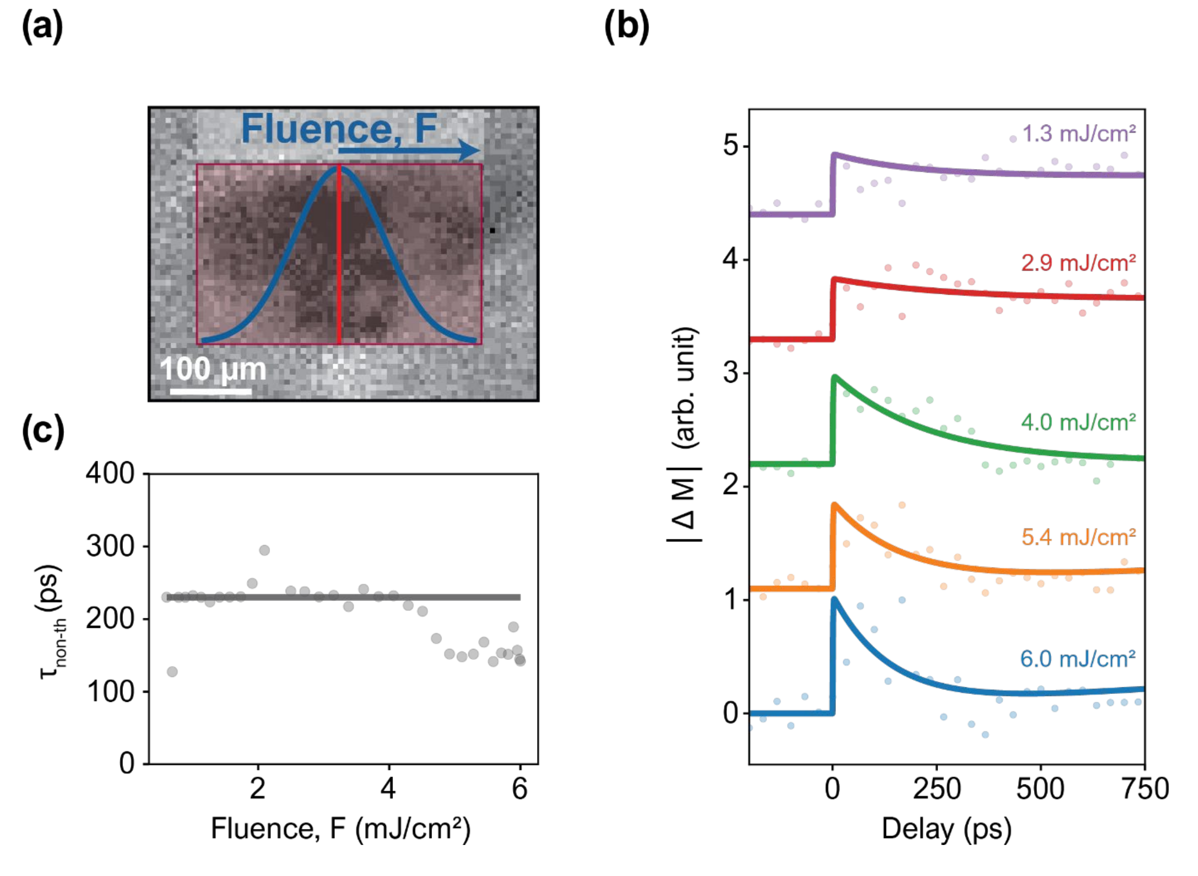

Figure S7| Micropulse fluence dependence through image slices. (a) An example of the method used to extract the dynamics at different fluences. A pixel wide slice (red vertical line) of a magneto-optical image is taken and compared to differing time delays. The horizontal position of the slice dictates the local fluence that was experienced on the sample through a Gaussian distribution (with the blue curve provided as a guide to the eye). **(b)** The magnetization dynamics given through the vertical slices in the magneto-optical images at different time delays. A three exponential fit is provided as a solid curve for each fluence. **(c)** The lifetime of the non-thermal process as a function of the determined fluence. A grey horizontal line is given as a guide to the eye.

Note S6. Extended Polarization Control

Regarding the polarization angles where ΔM is close to zero (e.g., around 165°), a coexistence of two largely homogeneous domains with opposite magnetization orientations is observed at early delays, consistent with the absence of an energetic preference for either state. This behaviour is consistent with our nonthermal model: at these polarizations, the coupling to the two energetically equivalent magnetization states is similar, leading to their equal population and thus a near-zero net magnetization. As shown in Fig. S8, over time the initially large nonthermal domains begin to fragment, giving rise to smaller ‘black’ and ‘white’ domains, which signals a gradual crossover toward a thermal, multidomain state.

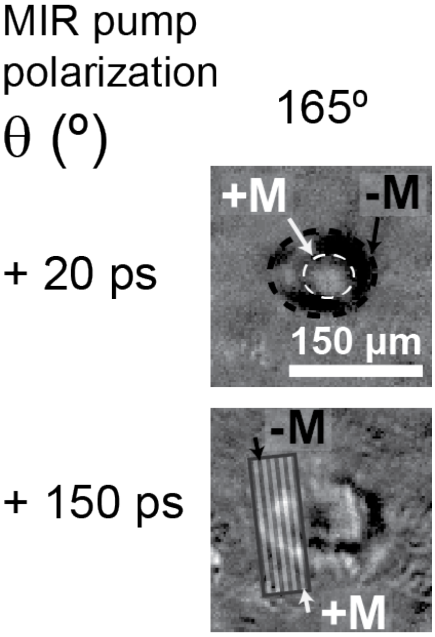

Figure S8 | Pump polarization dependence The pump polarization is here described as the angle θ between the crystallographic a-axis and the impulse light's polarization. Two sets of magneto-optical images were taken, one at a delay of 20 ps after the arrival of the pump beam and another at 150 ps. At 20 ps two homogeneous domain are highlighted. A stripe pattern that emerges at 150 ps is displayed.

When phonon pumping in $DyFeO_3$, coherent spin precession are additionally excited [3]. To understand how the pump polarization can affect these spin precessions, we performed additional tabletop stroboscopic pump–probe measurements to clarify which pathway governs the phonon-driven response. As shown in Fig. S9, the pump polarization modifies the amplitude of the spin precession, which shows some correlation with the polarization dependence of the phonon-induced magnetization. However, it does not reverse the precession phase. Therefore, while an anisotropic contribution from the precession may be present, it does not appear to set the state selection.

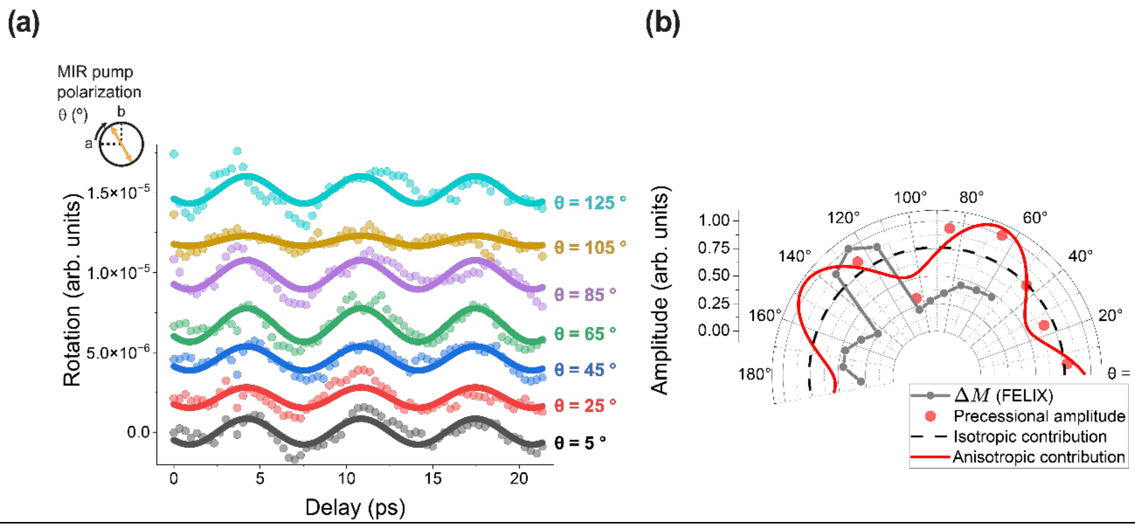

Figure S9 | Polarization dependent spin precession (a) The resulting pump induced spin precession observed through the magneto-optical time-resolved Faraday effect extracted after an exponential fit. The solid curves are sine wave fits performed along the relevant data points for a given pump polarization θ. **(b)** The amplitude of the sine fits performed in **(a)** are presented along with the given pump polarization θ. The isotropic contribution is displayed as the black curve, while an additional sine fit is given to highlight its anisotropic behavior. The sample was held at the AFM phase with a temperature of 34 K while an external magnetic field of 20 mT was applied. The pump wavelength used was 14 μm. Additionally, grey data points show the change in magnetization through the phonon-induced phase transition, where now an amplitude of 0 represents the black magnetization state and an amplitude of 1 represents the white magnetization state seen previously through magneto-optical imaging.

Note S7. Wavelength dependence between the macro- and micropulse

In Fig. S10B the wavelength dependence of the phonon-induced phase transition is presented for both the macropulse regime (Fig. S10A) as well as the micropulse regime (Fig. S10C). Both wavelength dependencies agree on the position of λ^{max}, where the resonance peaks at λ^{max} = 14 μm and λ^{max} = 18.5 μm for both regimes.

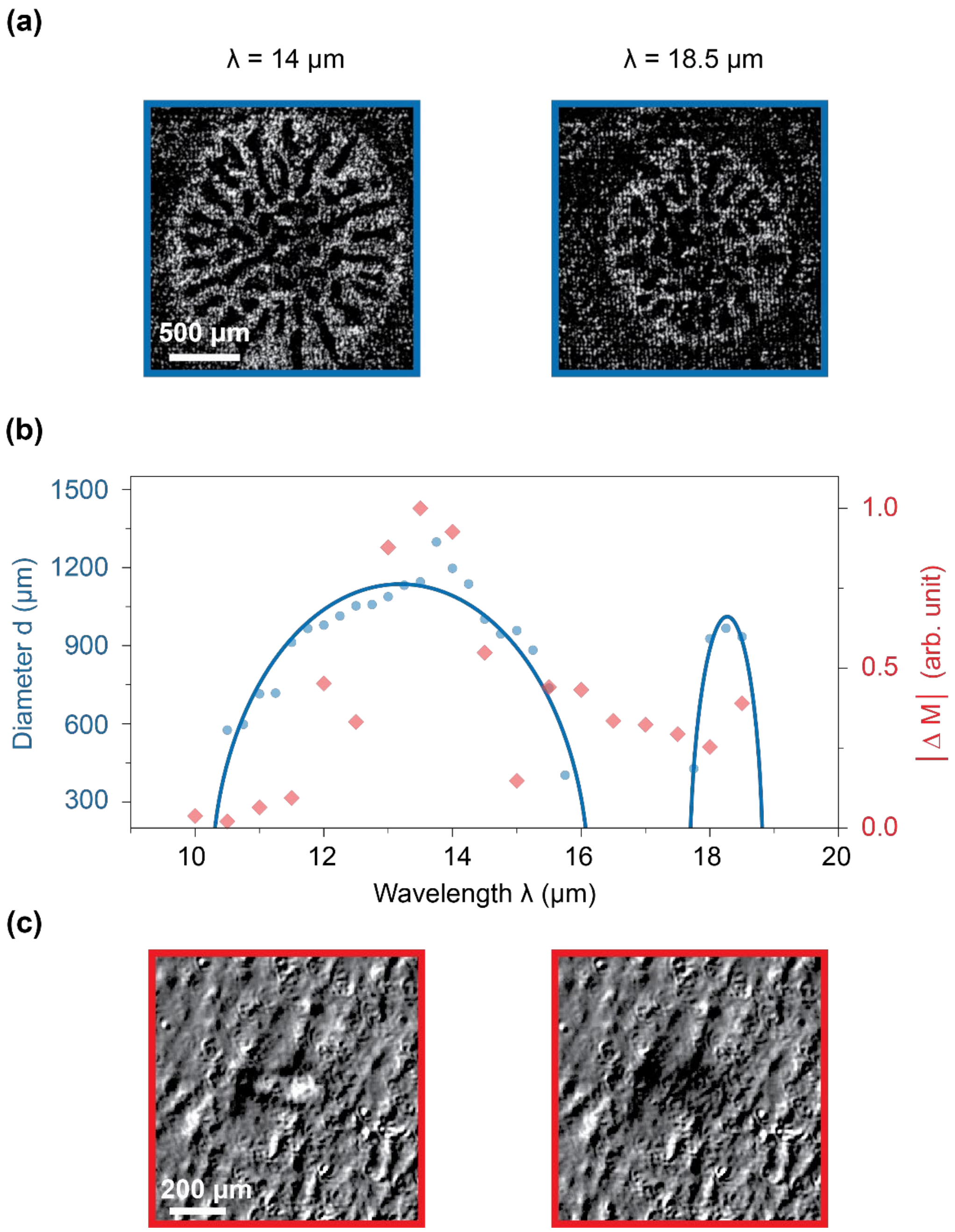

Figure S10 | Ultrafast dynamics induced by a single MIR micro-pulse at 18.5 µm. A comparison between the wavelength dependence using the macropulse (**(a)** and **(b)** left, blue dots), measured using the diameter of the switched spot, and the micropulse (**(c)** and **(b)** right, red diamonds), measured using the integrated intensity. The blue curve is the fitting procedure performed on the macropulse results. Two sets of magneto-optical (MO) images highlight the peak amplitude from the graphs at λ = 14 µm and λ = 18.5 µm. **(a)** The blue border signifies the macropulse regime while **(c)** the red border represents the micropulse regime. The micropulse MO images are taken 90 ps after the pump pulse.

Note S8. Ultrafast dynamics induced by a single MIR micro-pulse at 18.5 µm

While our results in the main text focused on the dynamics at 14 µm due to the stronger response, Fig. S11 shows that the ultrafast dynamics are similar to Fig. 3 for the phonon resonance found at 18.5 µm. The shape of the photo-excited spot appears to be highly elliptical as it forms, shown in Fig. S11a. This can be due to the shape of the elliptical

micropulse itself at this wavelength. Despite the shape of the spot, the dynamics remain largely unchanged, as Fig. S11B and Fig. S11C shows. The initial rise of the magnetization is on a similar timescale as seen in Fig. 4b at $\tau_{rise} = 7$ ps.

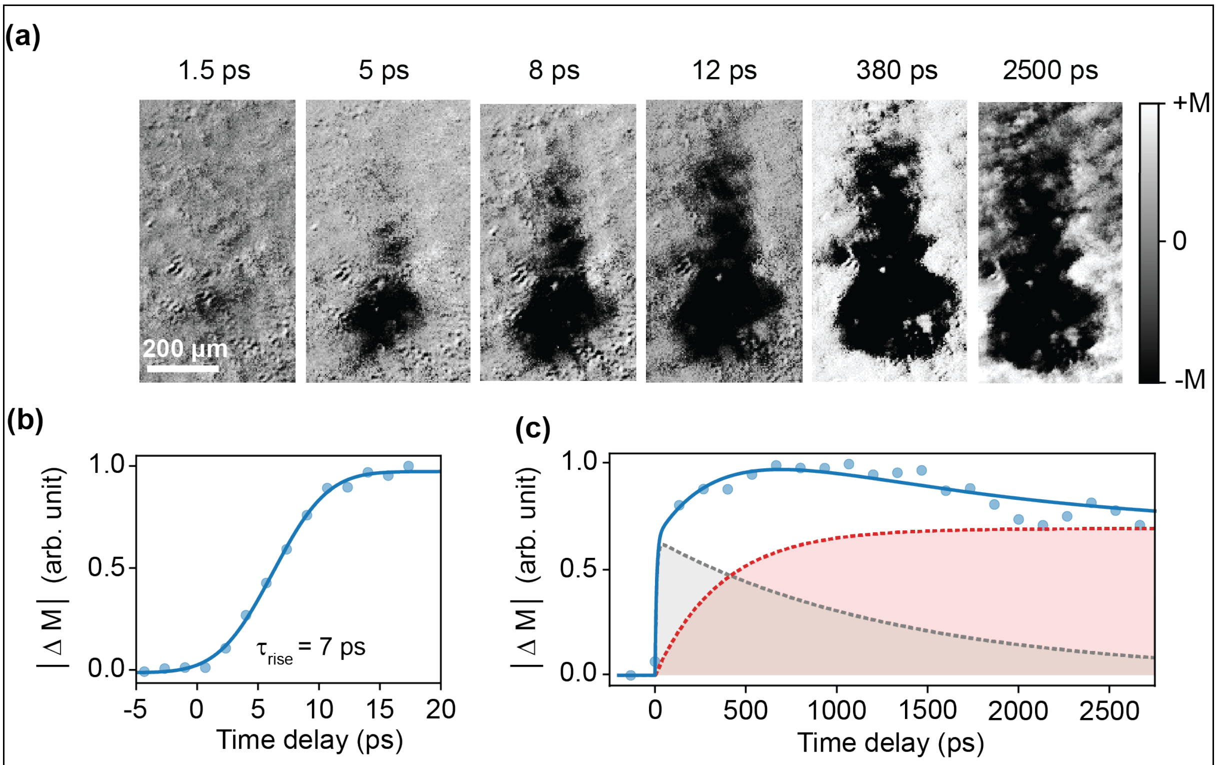

Figure S11 | Ultrafast dynamics induced by a single MIR micro-pulse at 18.5 µm. (a) Magneto-optical images are shown at different time delays to highlight the development of the Morin phase transition (PT) when pumping at 18.5 µm from an initial temperature of 46 K. **(b)** The magnetization dynamics are shown, where the normalized magnetization is plotted against the time delay in ps after the pump beam up to 20 ps. The solid line represents a fit using an error function. **(c)** The magnetization dynamics of the Morin PT is shown by displaying the normalized magnetization as a function of the time delay up to 2700 ps. The solid blue line in the graph is a fit with three exponentials representing the initial rise, exponential decay, and a slower exponential rise. The dotted red line is the part of the fit that highlights the thermal contribution, and the dotted gray line is the non-thermal contribution to the fit.

We performed the same fitting procedure for the dynamics up to 2.7 ns and found that we can identify the three processes seen in Fig. 3c: the initial fast rise, the exponential decay, and the slower exponential rise. The timescale for the exponential decay, however, is not on the same order as the slower exponential rise, suggesting that the nonthermal component remains present for the duration of the time interval measured. One reason for this discrepancy can be due to a lack of data points supporting this process in comparison to the results seen in Fig. 3.

References

1. Cui, R., Jiang, H., Du, Y., Xu, Y., Jia, Y., Sun, K., and Hao, X. (2024). Role of Dy *4f* electrons on magnetic coupling and reorientation in DyFeO3. J. Phys.: Condens. Matter *36*, 335501. https://doi.org/10.1088/1361-648X/ad49f9.

2. Hoogeboom, G.R., Kuschel, T., Bauer, G.E.W., Mostovoy, M.V., Kimel, A.V., and van Wees, B.J. (2021). Magnetic order of Dy^{3+} and Fe^{3+} moments in antiferromagnetic $DyFeO_3$ probed by spin Hall magnetoresistance and spin Seebeck effect. Phys. Rev. B *103*, 134406. https://doi.org/10.1103/PhysRevB.103.134406.

3. Afanasiev, D., Hortensius, J.R., Ivanov, B.A., Sasani, A., Bousquet, E., Blanter, Y.M., Mikhaylovskiy, R.V., Kimel, A.V., and Caviglia, A.D. (2021). Ultrafast control of magnetic interactions via light-driven phonons. Nat. Mater. *20*, 607–611. https://doi.org/10.1038/s41563-021-00922-7.

4. Balbashov, A.M., Volkov, A.A., Lebedev, S.P., Mukhin, A.A., and Prokhorov, A.S. (1985). High-frequency magnetic properties of dysprosium orthoferrite. Journal of Experimental and Theoretical Physics *88*, 974–987.
